# AhABI4s Negatively Regulate Salt-Stress Response in Peanut

**DOI:** 10.3389/fpls.2021.741641

**Published:** 2021-10-14

**Authors:** Lu Luo, Qian Wan, Kun Zhang, Xiurong Zhang, Ruijie Guo, Cai Wang, Chengchao Zheng, Fengzhen Liu, Zhaojun Ding, Yongshan Wan

**Affiliations:** ^1^State Key Laboratory of Crop Biology, Shandong Key Laboratory of Crop Biology, College of Agronomy, Shandong Agricultural University, Tai'an, China; ^2^Key Laboratory of Plant Development and Environmental Adaptation Biology, Ministry of Education, College of Life Sciences, Shandong University, Qingdao, China; ^3^State Key Laboratory of Crop Biology, Shandong Key Laboratory of Crop Biology, College of Life Sciences, Shandong Agricultural University, Tai'an, China

**Keywords:** peanut ABI4s, salt stress, transcriptome, quantitative proteome and phosphoproteome, downstream target, ion transporter/channel

## Abstract

Soil salinity is one of the major factors that limit the area of cultivable land and yield potential of crops. The ability of salt tolerance varies with plant species. Peanut (*Arachis hypogaea* L.) is a moderately salt-sensitive and economically important crop, however, their biological processes involved in salt-stress response remain unclear. In this study, we investigated the role of *A. hypogaea* L. ABSCISIC ACID INSENSITIVE 4s (*AhABI4s*) in salt tolerance and elucidated its mode of action in peanuts. The results showed that the downregulation of *AhABI4*s *via* whole plant virus-induced gene silencing has enhanced the survival rate, biomass accumulation, and root/shoot ratio of peanut seedlings in response to salt-stress. Transcriptomics, quantitative proteomics, and phosphoproteomic analyses were performed using *AhABI4*s-silenced and Mock plants. The expression pattern of 15,247 genes, 1,900 proteins, and 2,620 phosphorylation sites were affected by silencing of *AhABI4*s in peanut leaf and root after sodium chloride (NaCl) treatment. Among them, 63 potential downstream target genes of ABI4 changed consistently at both transcription and translation levels, and the protein/phosphorylation levels of 31 ion transporters/channels were also affected. Electrophoretic mobility shift assays (EMSA) showed that ABI4 was able to bind to the promoters of *HSP70, fructokinase* (*FRK*), and *pyruvate kinase* (*PK*) coding genes *in vitro*. In addition, we also detected a binding preference of *AhABI4* for CACT(G/T)GCA motif in the promoters of down-regulated genes in peanut leaf. Collectively, the potential downstream targets which were regulated at the levels of transcription and translation, binding preference, and *in vivo* phosphorylation sites that had been revealed in this study will provide new insight into the *AhABI4s*-mediated salt tolerance regulation mechanism in peanuts.

## Introduction

Soil salinity is a major factor restricting global agriculture development. Excessive soluble salt in the soil, mainly sodium (Na^+^) and chloride (Cl^−^) ions (Ismail et al., 2014; Chang et al., 2020), lead to both osmotic stress and ion stress during plant growth and development (Yang and Guo, 2018b). The osmotic stress not only compromises the ability to take water (Hasegawa et al., 2000) but also leads to rapid closure of stomata, which reduces the assimilation of carbon dioxide (CO_2_; Hedrich and Shabala, 2018). The ion stress caused by over accumulation of Na^+^ and Cl^−^ in plant cells is harmful to plant metabolism and the physicochemical properties of the cell wall (Munns and Tester, 2008; Cheeseman, 2013; Endler et al., 2015; Zhang et al., 2016). Both osmotic and ionic stress can promote secondary stress such as oxidative damage in plants (Genisel et al., 2014; Hazman et al., 2015; Li et al., 2015). Moreover, osmotic stress-induced stomatal closure and ion stress impair the photosynthetic machinery (Zhao et al., 2020), which is the major mechanism by which salt-stress inhibits plant growth (Bose et al., 2017). Peanut (*Arachis hypogaea* L.) is an economically important crop that furnishes protein and edible oil for human consumption and nutrition. To a great extent, peanut production is challenged by salt-stress because of the widely distributed saline-alkaline land in China (Sui et al., 2018). Therefore, it is necessary to study peanut salt-stress response and tolerance regulation mechanisms for key regulatory gene identification and breeding of salt tolerance cultivars.

Plant salt tolerance is a complex trait controlled by multiple genes or pathways, and the knowledge on salt-stress response and tolerance mechanisms mostly from works on the model plant *Arabidopsis thaliana* (van Zelm et al., 2020). Sensing and signaling are the crucial components of the salt-stress tolerance network (Yang and Guo, 2018b). The extracellular high concentration of Na^+^ and osmotic is first perceived by the membrane receptors/sensors, and further activate the salt-stress-induced signaling, especially salt-stress-response protein kinases and transcription factors (TFs) to induce expression of regulatory genes (Jiang et al., 2019; Zhao et al., 2020). Most TFs are involved in several biological processes or stress signaling pathways. Many studies have shown that several families of plant TFs modulate responses in the salt-stress signaling pathway, for instance, basic leucine zipper (bZIP), apetala/ethylene-responsive factor (AP2/ERF), basic helix-loop-helix (bHLH), MYB (v-myb avian myeloblastosis viral oncogene homolog), WRKY, and NAM, ATAF1,2, CUC2 (NAC) families (Fernando, 2020). Most of the TFs that have been implicated in salt-stress tolerance play positive roles, and the overexpression lines represent enhanced salt tolerance with high sensitivity to abscisic acid (ABA), less accumulation of Na^+^ and reactive oxygen species (ROS), or more protective compound content such as proline (Ayub et al., 2020; Fernando, 2020; Singh et al., 2021). A small amount of TFs belongs to WRKY, MYB, or AP2/ERF families, such as *PcWRKY33, ZmWRKY114*, and *ABA-INSENSITIVE 4 (ABI4)* are reported to play negative roles in salt-stress tolerance regulation in a different manner (Cai et al., 2017; Bao et al., 2018; Bo et al., 2020).

ABI4 is an evolutionary conserved AP2/ERF family TF and was first discovered in a genetic screen for ABA insensitive mutant (Finkelstein, 1994). The ABI4 protein consists of a CMIV-1 motif, an AP2/ERF domain, and an “ABI4 motif,” and binds to the promoter of target genes *via* the AP2/ERF domain (Wind et al., 2013). Several studies have proved that ABI4 recognizes *cis*-elements such as S-box (CACYKSCA), CCAC motif, or CE1 element (CACCG) in the promoter region to activate or repress gene expression (Chandrasekaran et al., 2020). The role of ABI4 in plant signaling transduction has been extensively studied in the past decades, and several important researches have been reported that ABI4 protein is a multi-dimensional regulator, including salt-stress response (Chandrasekaran et al., 2020). In *Arabidopsis*, the expression of *ABI4* is markedly induced by ABA and NaCl (Kong et al., 2013). During germination, salt-stress-induced expression of ABI4 promotes ROS production by directly enhancing *RbohD* expression, at the same time, ABI4 also impairs ROS scavenging by directly repressing *VTC2* transcription (Luo et al., 2020). ABI4 was reported to bind the promoter of sodium transporter coding gene *high affinity potassium transporter1;1 (HKT1;1)* and reduce its expression in root, whereas mutation of *ABI4* enhanced the expression of *HKT1;1* and contribute to *Arabidopsis* salt tolerance by reducing the accumulation of Na^+^ in the shoot (Shkolnik-Inbar et al., 2013). In addition, among the root-expressed genes affected by salt, the ABI4 binding element is highly frequent in the promoter of genes involved in transport processes (Shkolnik-Inbar et al., 2013), but how does ABI4 affect the expression of these genes, and whether translation or post-translational modification level of these transporters responses to salt-stress or not is still unknown.

Peanut (*A. hypogaea* L.) is an allotetraploid (2n = 4x = 40) with two subgenomes. Publication of wild type and cultivar genomic sequences of peanut has helped elucidate various biological processes in peanut *via* omics methods (Bertioli et al., 2016, 2019; Chen et al., 2016; Yin et al., 2018; Zhuang et al., 2019). In recent years, the identification of salt-stress response genes in peanuts was profiled mainly by transcriptome analysis. Pathogenesis-related 10 (PR10), small GTP-binding protein (AhRab7), ω-3 fatty acid desaturase, and bZIP TFs may enable peanut to adapt to or recover from salt-stress (Jain et al., 2011; Sui et al., 2017, 2018; Cui et al., 2018; Wang et al., 2019). However, the transformation efficiency of peanuts was usually low and cultivar-dependent (Krishna et al., 2015; Karthik et al., 2018). Moreover, this process is technically difficult and obtaining a stable transgenic plant is time-consuming (Lv et al., 2020; Qiao et al., 2021); it might also lead to somaclonal variations (Mayavan et al., 2015; Lv et al., 2020). These disadvantages restricted gene function studies in peanuts greatly. In recent years, virus-induced gene silencing (VIGS) has been used as a powerful functional genomics tool to assess gene function for species not amenable to stable genetic transformation. The application in peanuts has also been reported (Xu et al., 2017). The whole-plant VIGS is mediated by vacuum infiltration, and the silence effect can be detected in both leaves and roots, as well as in reproductive organs (Qu et al., 2012). As indicated above, VIGS can be considered as an effective reverse-genetic strategy for peanut gene function analysis and regulatory mechanism study.

In this study, two ABI4-encoding genes were cloned from peanut cultivar Fenghua2 (Fh2, Spanish type) and silenced by the whole-plant VIGS method. The *AhABI4*s-silenced plants showed a significantly higher survival rate, biomass accumulation, and root/shoot ratio than those in Mock plants under salt-stress. According to the transcriptome analysis, there were differences in the ABI4-binding element in promoters of ABI4-affected salt responding genes. The CACT(G/T)GCA type element was only enriched in promoters of downregulated leaf-expressed genes, while the enriched element was CACTTGCA in promoters of root-expressed and upregulated leaf-expressed genes. In peanut leaves and roots, 63 probable direct downstream targets of *AhABI4s* under salt-stress were identified *via* transcriptome and proteome analysis. Among them, ABI4 protein could bind to the promoters of HSP70-, fructokinase-, and pyruvate kinase-encoding gene *in vitro*. Moreover, the silence of *AhABI4*s affected protein level, phosphorylation sites, or phosphorylation level of 31 ion transporters/channels. It is clarified in this study that *AhABI4*s negatively regulate salt tolerance and biomass accumulation under salt-stress in peanut seedlings. The multi-omics data displayed here not only provided an important basis for functional analysis of *AhABI4*s in salt-stress tolerance but also furnished valuable references for protein phosphorylation and function analysis under salt-stress.

## Materials and Methods

### Plant Materials and Growth Conditions

Mature seeds of *A. hypogaea* L. Cultivar Fh2 (bred by Shandong Agriculture University, and stored by our group) were used for testing. Dry seeds similar in size were surface-sterilized with 0.1% (v/v) H_2_O_2_ for 12 h. Then, the seeds were rinsed by sterilizing deionized water three times, 1 min each. The seeds were then set on wet degreasing cotton in seedling disks (Longji Plastic Co. Ltd., Taizhou, China) that were incubated at 26°C in the dark for 3 days. The germinated seeds were then exposed to long-day (LD) conditions (16 h light/ 8 h dark). Seedlings with two functional leaves were then transplanted in a hydroponic-box and cultured with 1/5 Hoagland's (Hope Bio-Technology Co., Ltd., Qingdao, China) nutrient solution (Duarte et al., 2015; He et al., 2017).

### Homology Cloning of Peanut ABI4-Encoding Genes

To identify the conserved fragment of *AhABI4*, gene-specific primers were designed with Primer Premier v.5 (PREMIER Biosoft International, San Francisco, CA, USA). Primary roots of 5-day seedlings were harvested for total RNA extraction and gene cloning. The PCR program was as follows: 94°C for 5 min; followed by 35 cycles of 94°C for 30 s, 55°C for 30 s, 72°C for 40 s; and then 72°C for 5 min. The products were separated on 3% (w/v) agarose. Primers of 3' and 5' rapid amplification of cDNA ends (RACE) were designed according to the sequence of the open reading frame (ORF) region of *AhABI4* (Supplementary Table 1). The 5' RACE was executed with 5' RACE System v.2 (Invitrogen, Carlsbad, CA, USA) following the instructions of the manufacturer. First-round PCR was performed according to the following program: 94°C for 5 min; 94°C for 30 s, 48°C for 30 s, 72°C for 40s, 35 cycles; and then 72°C for 5 min. The products were diluted to the appropriate concentration and used in the second PCR round, according to the following program: 94°C for 5 min; followed by 35 cycles of 94°C for 30 s, 55°C for 30 s, 72°C for 40 s; and then 72°C for 5 min. Negative controls were simultaneously run using signal primers. Target fragments were purified with a gel extraction kit (Omega Bio-Tek Inc., Norcross, GA, USA), and subcloned into pEasy T1 simple cloning vector (Transgen Biotech, Beijing, China). Recombinant clones were screened by bacterial colony PCR according to the following program: 94°C for 10 min; followed by 35 cycles of 94°C for 30 s, 55°C for 30 s, 72°C for 40 s; and then 72°C for 5 min. Primer synthesis and DNA sequencing were conducted by Sangon Biotech, Shanghai, China.

### Whole Plant Virus-Induced Gene Silencing (VIGS) of *AhABI4*s

The psTRV1 (synthetic Tobacco Rattle Virus 1) and psTRV2 plasmids (Qu et al., 2012) were used in the VIGS assays. The psTRV1, psTRV2, and psTRV2:*AhABI4* vectors were transformed into *Agrobacterium tumefaciens* GV3101 competent cells by freeze and thaw methods (Máximo et al., 2017), respectively. Positive clones were selected by bacterial colony PCR. A single colony was inoculated into 10 ml of Luria-Bertani (LB) liquid medium (Hope Bio-Technology Co., Ltd., Qingdao, China) supplemented with 50 mg/L kanamycin (Sangon Biotech, Shanghai, China) and 20 mg/L rifampicin (Sangon Biotech, Shanghai, China) and incubated in a shaking incubator until OD_600_ = 0.6. For secondary activation, 2 ml culture was inoculated into 100 ml LB liquid medium supplemented with 50 mg/L kanamycin, 20 mg/L rifampicin, 10 mM methyltransferases (MES), and 20 μM acetylsyringone and grown overnight until OD_600_ reached 1–1.5. Agrobacteria were collected by centrifugation at 6,000 rpm and room temperature for 10 min, resuspended in a monomethylamine (MMA) solution [10 mM MES, 10 mM magnesium chloride (MgCl_2_), and 20 μM acetylsyringone] to a final concentration of OD_600_ = 1 and maintained at room temperature without shaking for 3 h. For vacuum infiltration, 5-day seedlings were immersed in the suspension, subjected to 0.07-0.08 MPa vacuum for 50 s, and slowly released from the vacuum. The infiltrated seedlings were maintained in the dark for 16 h and subjected to a 16 h light/8 h dark photoperiod at 25°C. At least 150 Mock and silenced plant seedlings each were vacuum infiltrated for every independent biological replicate. A single seedling was considered an independent transformation event in the subsequent experiments. To evaluate the expression level of *AhABI4*s after VIGS treatment, newly grown leaves from each plant were harvested and considered as a single sample.

### Stress Treatment and Sample Selection

To analyze *AhABI4* expression patterns, 2-week seedlings were treated with nutrient solution containing 200 mM NaCl, and *AhABI4* expression levels were measured at 0, 6, 12, 18, and 24 h. For the salt tolerance assay, the seedlings were transferred to a nutrient solution containing 200 mM NaCl and left to grow for another 2 weeks. The roots were then washed, and the seedlings were recovered in a normal nutrient solution. For leaf disc assay, leaf disks with 6 mm in diameter were punched out of leaves grown after infiltrating. The detached leaves were subjected to 100, 150, or 200 mM NaCl and 0.9% (w/v) NaCl served as the control.

For the expression pattern analyses, tissues from five individual seedlings were selected and pooled in a single sample. For the transcriptome, proteome, and phosphoproteome analyses, Mock, *AhABI4*s-silenced leaf and root samples either untreated or exposed to 200 mm NaCl were collected and frozen in liquid nitrogen (LN). Each sample comprised 30 individual seedlings, which were mixed thoroughly and divided into four sections for the above analyses and storage. Before the NaCl treatment, the *ABI4* mRNA levels in each line were detected using semi-quantitative PCR.

### RNA Isolation, Reverse Transcription, Qrt-PCR, and Illumina Sequencing

Total RNA was isolated with a Quick RNA isolation kit (Waryong, Beijing, China) following the instructions of the manufacturer. Samples were quantified by NanoDrop 2000 microvolume spectrophotometry (Thermo Fisher Scientific, Wilmington, DE, USA) and 1 μg total RNA was reverse transcribed with an Advantage RT-for-PCR kit (Takara Bio, Dalian, China) or PrimeScript RT reagent Kit with DNA Eraser (Takara Bio, Dalian, China) following the protocols of the manufacturers. Total RNA and cDNA were stored at −80°C and −20°C, respectively.

Quantitative RT-PCR was performed on an ABI StepOne Real-Time PCR Systems (Thermo Fisher Scientific, Wilmington, DE, USA) using SYBR *Premix Ex Taq* (Perfect Real Time, Takara Bio, Dalian, China). Three technical replicates were included in each biological replicate and three biological replicates were performed. Primers are listed in Supplementary Table 1.

The RNA library was prepared with a NEBNext Ultra RNA library prep kit for Illumina (New England Biolabs Inc., Ipswich, MA, USA). Paired-end sequencing was conducted in the HiSeq X Ten system (Illumina, San Diego, CA, USA).

### Protein Extraction, Trypsin Digestion, and TMT Labeling

Samples were ground in LN and sonicated three times in lysis buffer on ice with a high-intensity ultrasonic processor (Scientz, Ningbo, China). An equal volume of Tris-saturated phenol (pH 8) was added and vortexed. After centrifugation at 5,000 × *g* at 4°C for 10 min, the upper phenol phase was collected. Proteins were precipitated by adding four volumes of ammonium sulfate-saturated methanol and incubated at −20°C for 6 h. After centrifugation at 4°C for 10 min, the supernatant was discarded. The remaining precipitate was washed with ice-cold methanol once, followed by ice-cold acetone three times. The protein was redissolved in 8 M urea and the protein concentration was determined with a bicinchoninic acid (BCA) protein assay kit (Pierce, Bonn, Germany) according to the instructions of the manufacturer.

For digestion, the protein solution was reduced with 5 mM dithiothreitol at 56°C for 30 min and alkylated with 11 mM iodoacetamide at room temperature in the dark for 15 min. The protein sample was then diluted by adding 100 mM triethylammonium bicarbonate (TEAB) to a urea concentration less than 2 M. Trypsin was added at a 1:50 (w/w) trypsin: protein ratio for the first overnight digestion, and at a 1:100 (w/w) trypsin: protein ratio for the second 4 h digestion. The peptide was then desalted in a Strata X C18 SPE column (Phenomenex, Torrance, CA, USA) and vacuum-dried. The peptide was reconstituted in.5 M TEAB and processed according to the TMT labeling kit protocol (Thermo Fisher Scientific, Wilmington, DE, USA).

### Biomaterial-Based PTM Enrichment and LC-MS/MS Analysis

For the phosphoproteome analysis, the peptide mixtures were first incubated with immobilized metal affinity chromatography (IMAC) microsphere suspension. IMAC microspheres with enriched phosphopeptides were collected by centrifugation and the supernatant was removed. To remove nonspecifically adsorbed peptides, the IMAC microspheres were sequentially washed with 50% (v/v) acetonitrile/6% (v/v) trifluoroacetic acid (TFA) and 30% (v/v) acetonitrile/0.1% (v/v) TFA. To elute the enriched phosphopeptides from the IMAC microspheres, elution buffer containing 10% (w/v) ammonium hydroxide (NH_4_OH) was added and the enriched phosphopeptides were eluted by vibration. The supernatant containing the phosphopeptides was collected and lyophilized for liquid chromatography with tandem mass spectrometry (LC-MS/MS) analysis.

The tryptic peptides were dissolved in 0.1% (v/v) formic acid (solvent A) and directly loaded onto a homemade reversed-phase analytical column (15-cm length, 75 μm i.d.). The gradient was comprised of an increase from 6 to 23% solvent B (0.1% (v/v) formic acid in 98% (v/v) acetonitrile) over 26 min, a 23–35% increase in solvent B over 8 min, an increase of solvent B up to 80% in 3 min, and holding at 80% for 3 min. The flow rate was a constant 400 ml/min. The apparatus consisted of an EASY-nLC 1000 UPLC system (Thermo Fisher Scientific, Wilmington, DE, USA) coupled to a Q-Exactive^TM^ Plus (Thermo Fisher Scientific, Wilmington, DE, USA) combined with UPLC.

### Database Search and Data Analysis

Raw data were first filtered and clean data were aligned to the reference genome (https://www.peanutbase.org/peanut_genome, *Arachis hypogaea* cv. Tifrunner). The MS/MS data were processed using the Maxquant search engine v.1.5.2.8 (Max Planck Institute of Biochemistry, Munich, Germany). Tandem mass spectra were searched against the transcriptome database concatenated with reverse decoy database. Trypsin/P was specified as a cleavage enzyme allowing up to four missing cleavages. The mass tolerances for the precursor ions were set to 20 ppm for the first search and 5 ppm for the main search. The mass tolerance for the fragment ions was set to 0.02 Da. Carbamidomethyl on Cys and was specified as a fixed modification, while Met oxidation and *N*-terminal and phosphor_STY (Ser, Thr, and Tyr) acetylation were specified as variable modifications. The false discovery rate (FDR) was adjusted to <1% and the minimum score for the modified peptides was set >40.

Genome sequences of the peanut cultivar were downloaded from PeanutBase (https://www.peanutbase.org/). Genome sequences of 17 other plant species were downloaded from Ensembl Plants (http://plants.ensembl.org/index.html). The eight-residue motif “LRPLLPRP” served as the query sequence for BLAST searches through each genome. The online tool “find individual motif occurrences (FIMO)” (Grant et al., 2011) in MEME Suite v. 5.3.1 (https://meme-suite.org/meme/tools/fimo) was used to scan the ABI4-binding motif S-box in the promoter region. TBtools (Chengjie Chen, Guang Zhou, China) were used to extract the promoter sequence, perform Venn analyses, and plot heatmaps (Chen et al., 2020).

Data from three independent experiments were processed by Microsoft office Excel 2019 software (Microsoft Corporation, Redmond, USA). Significant analysis was performed by GraphPad Prism v.8.0.2 (GraphPad Software Inc., La Jolla, CA, USA) by Student's *t*-tests (*P* < 0.05).

### Prokaryotic Expression, Purification of Recombination of ABI4 Protein and EMSA

The ABI4 coding sequence without stop codon was amplified from *A. thaliana* ecotype Col-0 plants and cloned into a *pET32a* vector with a 7 × His tag. Recombinant ABI4 was expressed in *Escherichia coli* BL21 and purified with His Sepharose beads (Sigma-Aldrich Corp., St. Louis, MO, USA). Electrophoretic mobility shift assay (EMSA) was performed with a Lightshift chemiluminescent EMSA kit (Thermo Fisher Scientific, Wilmington, DE, USA). The biotin-labeled primers listed in Supplementary Table 1 served as probes.

## Results

### *AhABI4*s Negatively Regulate Salt Tolerance in Peanut

The ABI4 motif, also called the LPR motif, is found exclusively in the ABI4 protein (Gregorio et al., 2014). A genome-wide analysis disclosed that there has only one copy of the ABI4 motif in one set of chromosomes in most plants (Supplementary Table 2). According to the published genome sequence of the diploid ancestors of cultivated peanut in PeanutBase (Bertioli et al., 2016), sequence-specific primers AhABI4-F/R (Supplementary Table 1) were designed to clone the Open Reading Frame (ORF) region of ABI4 genes from peanut cultivar Fenghua 2 (Fh 2, Spanish type). Sequences of 1,092 and 1,074 bp in length were obtained. The amino acid sequences of the two potential AhABI4s resembled the published plant ABI4s and both contained the “ABI4 motif” (Supplementary Figure 1A, Supplementary Table 3). RACE experiment was performed to get a full-length cDNA sequence of the genes. According to their homology to *ABI4*s present in *A. duranensis* and *A. ipaensis*, which contribute to the subgenome A and B of modern cultivated peanut, the newly cloned genes were named *AhABI4A* (GenBank accession No. MN088829) and *AhABI4B* (GenBank accession No. MN088830), respectively (Supplementary Figure 1B, Supplementary Table 4).

Semi-quantitative RT-PCR was performed to detect the expression levels of AhABI4 coding genes under salt-stress. As shown in Supplementary Figures 1C–E, the expression levels of *AhABI4*s were significantly upregulated in the tap and lateral roots after 3 h treatment under salt-stress, while the high peak of expression level in leaf was present after 6 h treatment with salt-stress. *AhABI4*s exhibited higher expression levels in the germinating seeds and embryos than that in the vegetative organs (Supplementary Figures 1F,G). These results showed that *AhABI4*s display similar expression patterns with other reported ABI4 coding genes in plants.

**Figure 1:**
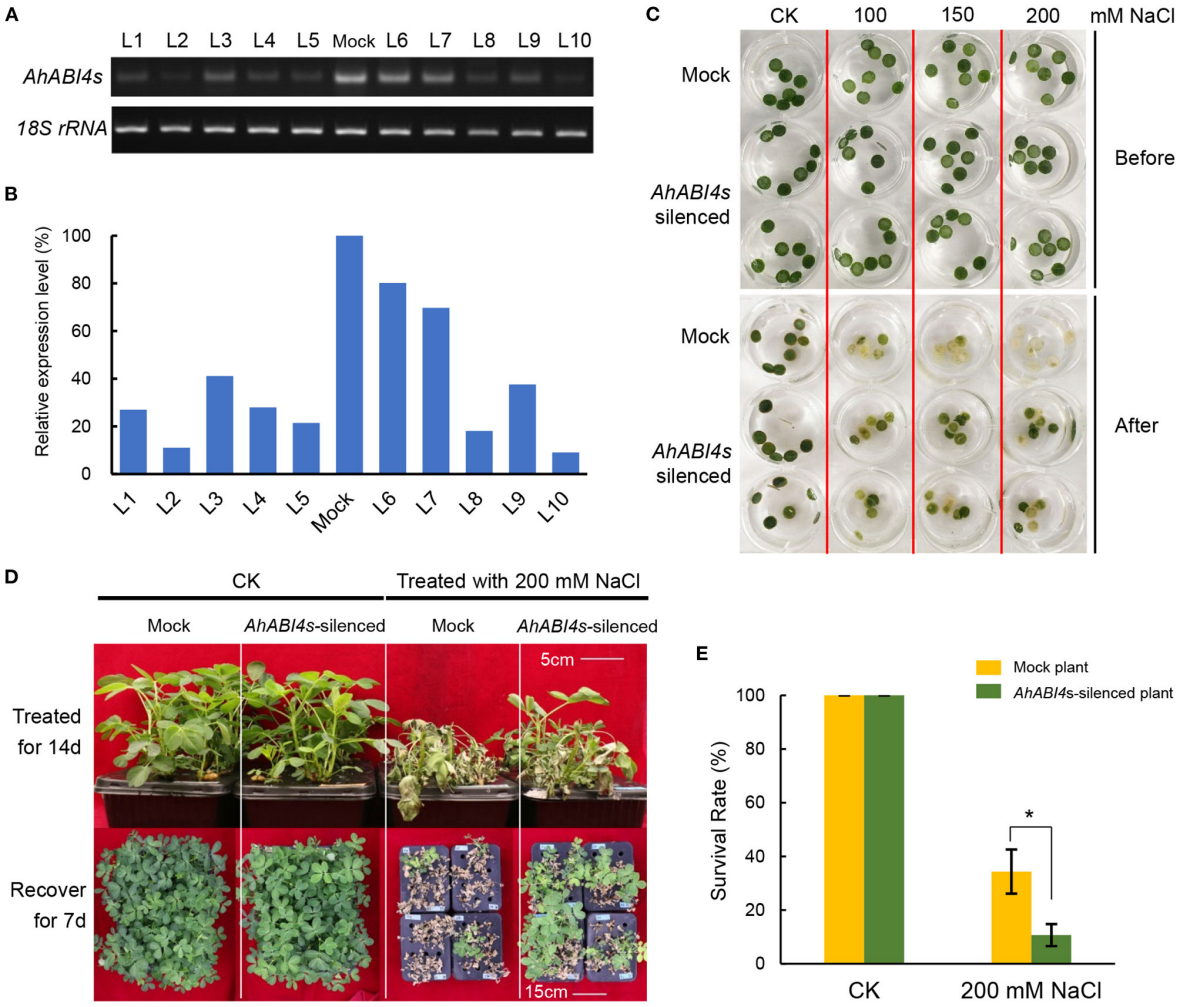
Phenotype analysis of *AhABI4*s-silenced lines under salt-stress. **(A,B)** Detection of *AhABI4* mRNA level at 7 d after inoculation. Target product quantity was calculated by gray scanning in ImageJ (NIH, Bethesda, MD, USA). The relative *AhABI4*s expression level in *AhABI4*s-silenced plants was compared against that in Mock plants. **(C)** Leaf disc assay under salt-stress. Two or three discs were punched from each seedling depending on leaf size and quantity. More than 20 seedlings were tested in each replicate. **(D)** Phenotypes of Mock and *AhABI4*s-silenced seedlings after the 14-day salt-stress treatment and 7-day recovery. **(E)** Survival rates of Mock and *AhABI4*s-silenced seedlings after the 7-day recovery with or without salt-stress treatment. At least 100 seedlings were tested per treatment and three independent experiments were performed. The student's *t*-test was performed to detect significant differences in survival rate between Mock and *AhABI4*s-silenced plants. *P < 0.05.

Whole plant VIGS (Supplementary Figure 2) was performed according to the previously reported study (Ye et al., 2009). A 786-bp fragment (for primers, refer to Supplementary Table 1) from the *phytoene desaturase* (PDS) gene in peanut (arahy.M5MKEZ) was used to determine if the VIGS system can work in peanuts. The mRNA levels of the PDS-coding gene in *AhPDS*-silenced lines declined significantly compared with that of the Mock plant (Supplementary Figure 3). To construct the psTRV2:*AhABI4* vector used in VIGS, a 328-bp fragment downstream of the AP2 domain (Supplementary Figure 1H, I-fragment; Supplementary Table 1) was cloned and recombined to linearized psTRV2 vector. Plants vacuum infiltrated with an *Agrobacterium* mixture containing psTRV1 and psTRV2 empty vectors were used as negative controls. To assay the validity and efficiency of VIGS, newly grown young leaves were harvested to detect their *AhABI4* expression levels at 7-days post-inoculation. Full-length ORFs were amplified with primers *AhABI4*-F and R (Supplementary Table 1) to determine the relative content of remaining *AhABI4*s mRNA. As shown in Figures 1A,B, we found a significant decline of *AhABI4*s mRNA level in silenced plants compared with Mock plants.

To further analyses, leaf disc assays were performed. Mock and *AhABI4*s-silenced lines were exposed to 1/5 Hoagland's nutrient solution with or without extra NaCl. After the 6-day treatment, the degree of bleaching in leaf discs of *AhABI4*s-silenced lines was not as remarkable as that of Mock plants (Figure 1C). Hydroponically grown seedlings were treated with 200 mM NaCl. Those cultured under normal conditions grew uniformly whereas both Mock and *AhABI4*s-silenced plants subjected to salt-stress presented with wilting after the 14-day treatment (Figure 1D, top). All seedlings were then exposed to 1/5 Hoagland's for recovery. After 7 days, 34.33% of the *AhABI4*s-silenced plants had survived whereas only 10.66% of the Mock plants remained alive (Figures 1D,E). Furthermore, biomass accumulation and root/shoot ratio were significantly higher in the *AhABI4*s-silenced plants than those in the Mock plants (Table 1). Hence, the decline of *AhABI4*s mRNA level improved the salt tolerance of the peanut cultivar by improving its relative survival and growth rates.

**Table 1:** Effect of silencing of *AhABI4*s on peanut seedlings growth under salt-stress.

**Time**	**Lines**	**Biomass (g)**		**Root/shoot ratio**	**Main stem height (cm)**		
		**Control**	**Salt-stress**		**Control**	**Salt-stress**	**Relative**
2d	Mock	0.46 ± 0.04	0.32 ± 0.01	0.19 ± 0.02	5.24 ± 0.26	3.83 ± 0.04	0.73
	*AhABI4*s-Scienced	0.43 ± 0.02	0.39 ± 0.01^**^	0.24 ± 0.01^*^	4.51 ± 0.12	4.54 ± 0.09	1.00
4d	Mock	0.62 ± 0.02	0.50 ± 0.02	0.26 ± 0.02	6.11 ± 0.09	4.88 ± 0.12	0.80
	*AhABI4*s-Scienced	0.64 ± 0.03	0.57 ± 0.01^*^	0.23 ± 0.04	6.03 ± 0.21	5.34 ± 0.14	0.89
6d	Mock	0.81 ± 0.03	0.57 ± 0.03	0.19 ± 0.02	8.58 ± 0.25	5.22 ± 0.04	0.61
	*AhABI4*s-Scienced	0.79 ± 0.02	0.62 ± 0.01^**^	0.23 ± 0.01^*^	8.33 ± 0.09	6.00 ± 0.12	0.72

*Three replicates were measured for each treatment*.

*,** *Indicated significant and extremely significant differences*.

### Characterization of Transcriptome of Peanut Under Salt-Stress

**Figure 2:**
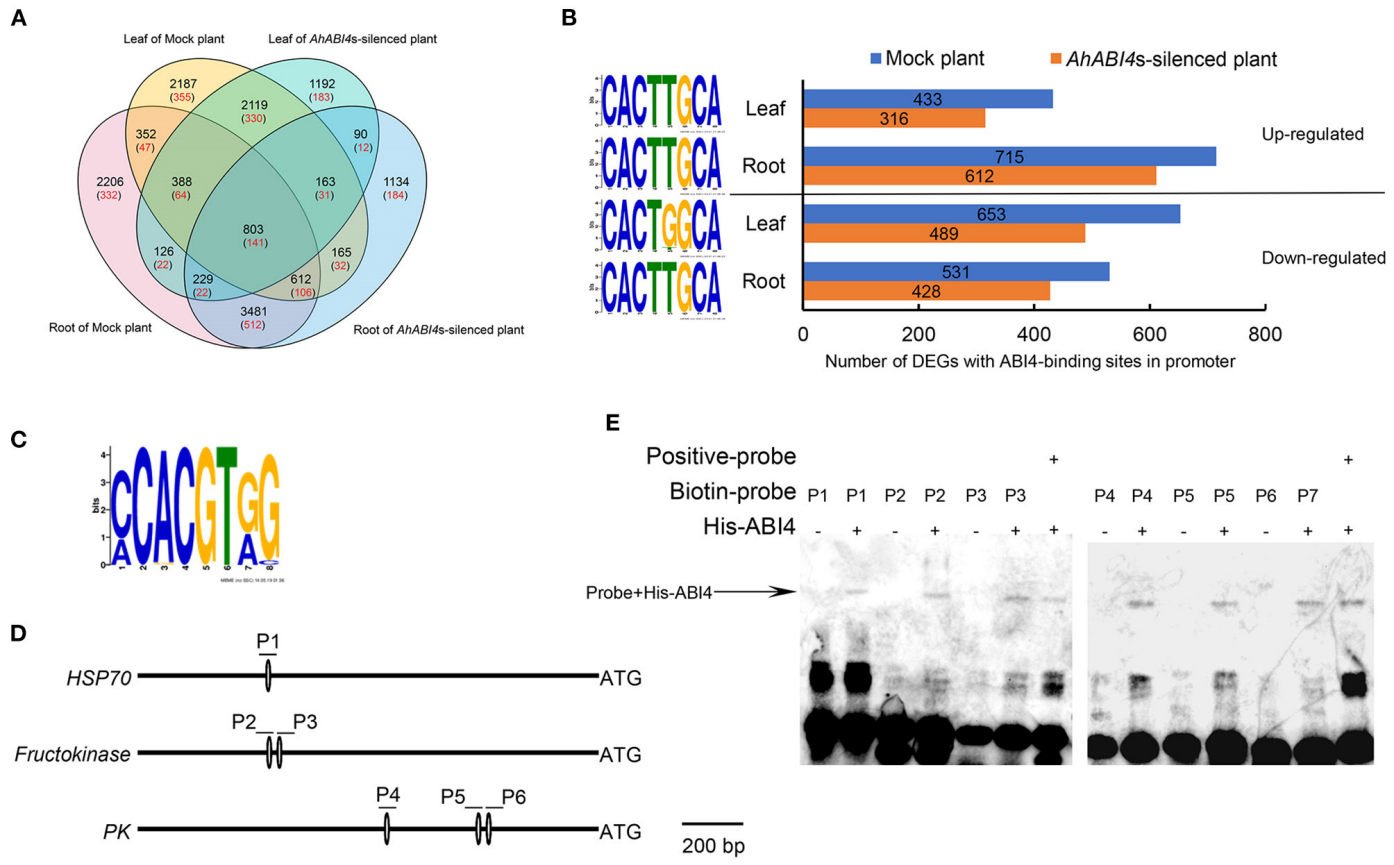
Analysis of potential downstream targets of ABI4 protein. **(A)** Venn analysis of all differentially expressed genes (DEGs; black) and differentially expressed putative downstream target of ABI4 (red) among samples. **(B)** Regulation type of DEGs with ABI4-binding S-box in their promoter region (right). The sequence of S-box in corresponding DEGs and tissues (left). **(C)** ABI4-binding motif in 63 putative downstream ABI4 targets under salt-stress. **(D)** Schematic diagrams showing ABI4-binding motif in promoter regions of *HSP70, fructokinase*, and *pyruvate kinase* genes. P1–P6 represent six pairs of probes containing ABI4-binding motifs. **(E)** EMSA showing ABI4 protein binding to promoters of *HSP70, fructokinase*, and *pyruvate kinase* genes.

To uncover the molecular mechanisms regulated directly by *AhABI4*s in peanuts under salt-stress, we performed RNA-seq analysis to quantify the transcriptome in both leaf and root of Mock and *AhABI4*s-silenced plants (Supplementary Figure 2). We calculated the normalized read counts (fragments per kilobase of transcript sequence per million base pairs sequenced, FPKM) of each gene and quantified 51,605 genes in all eight samples (Supplementary Table 5). The absolute value of log 2-fold change of 15,247 genes are larger than 1 after salt-stress treatment (Figure 2A, black number, Supplementary Table 5), which were defined as differentially expressed genes (DEGs).

A 1,500-bp region upstream of the initiation codon ATG in each of the aforementioned genes was extracted from the whole genome sequence of peanut cultivar *Tifrunner* (Bertioli et al., 2019). The S-box was found in the promoters of 2,353 DEGs (Figure 2A, red numbers) that are putative downstream targets of AhABI4 protein. Gene ontology (GO) enrichment indicated that the putative downstream targets participate in several stress-related biological processes (Supplementary Figure 4). Upregulated and downregulated downstream targets were detected in all tissues and relatively more DEGs were identified in Mock plants (Figure 2B). Enrichment of the S-box revealed that the S-box in the promoters of all upregulated and downregulated DEGs in the roots shared high sequence similarity (CACTTGCA), while the fifth position of the motif in the promoters of the downregulated genes in the leaf preferred G (Figure 2B). The expression patterns of the 2,353 DEGs were compared with those of their corresponding proteins. There were 63 genes (23 downregulated and 40 upregulated) which shared similar tissue expression patterns and transcription and translation changes (Supplementary Figure 5). CCAC motifs and S-boxes were identified in the promoters of these genes (Figure 2C, Supplementary Figure 5).

We then conducted EMSA to confirm the above prediction. His-tag recombinant ABI4 protein (AT2G40220) was expressed according to a previously reported method (Huang et al., 2017). Using the CE1 element of *ARR6* in *Arabidopsis* as a positive control (Huang et al., 2017), the ABI4 protein could bind to the S-box in the promoters of arahy.3X8NRX (*HSP70*, P1), arahy.FS0NK9 (probable *fructokinase 7*, P2, and P3), and arahy.SF2YP5 (*pyruvate kinase*, P4-P6) *in vitro* (Figures 2D,E). These results indicated that *AhABI4*s perform as either transcription activators or repressors in peanuts under salt-stress, and the differences in the sequence of S-box may play an important role in the determination of the binding specificity of ABI4 proteins.

### Characterization of Proteome and Phosphoproteome of Peanut Under Salt-Stress

**Figure 3:**
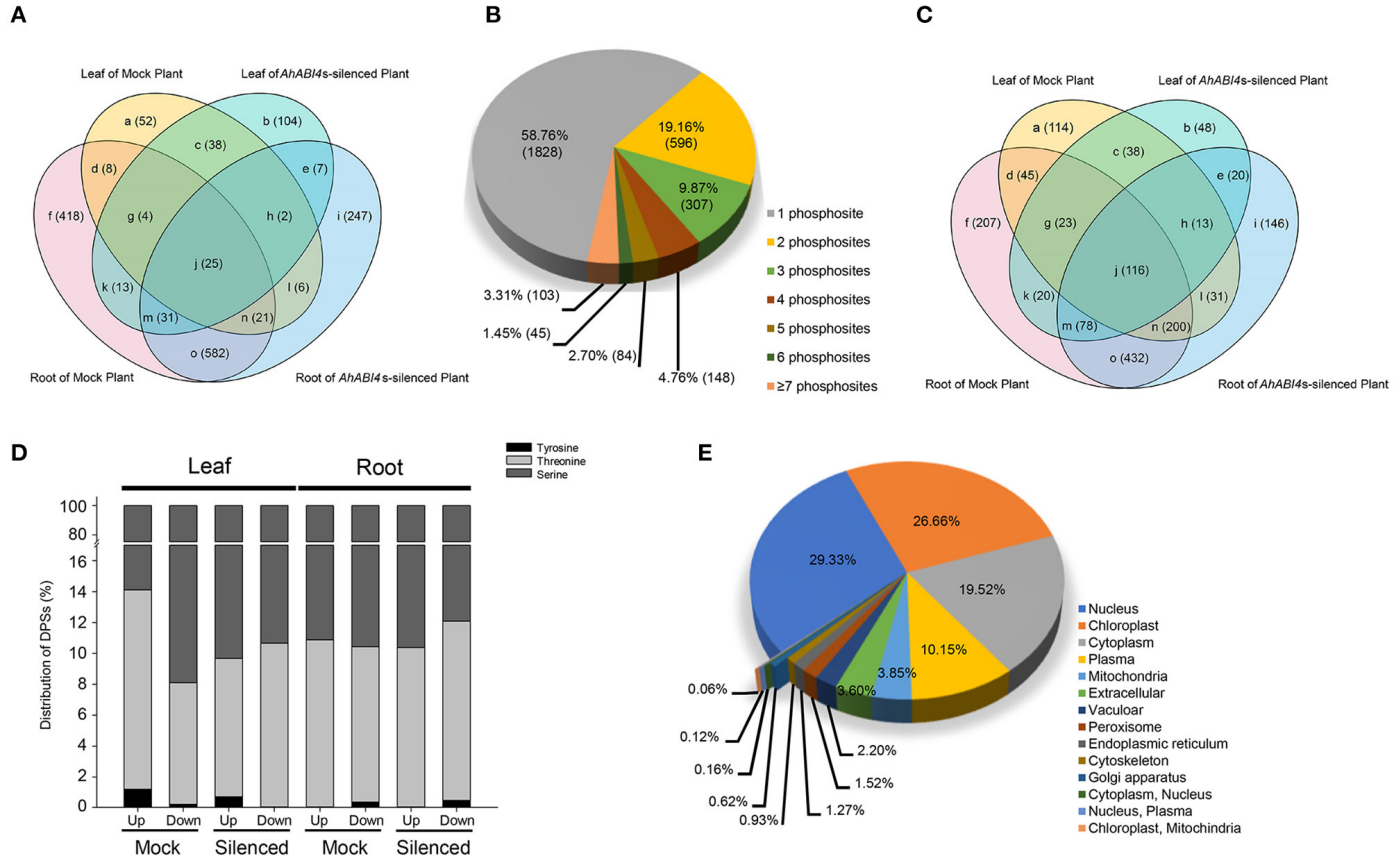
Characteristics of the proteomic and phosphoproteomic data in Mock and *AhABI4*s-silenced peanut leaf and root. **(A)** Overlap of the DEPs identified in different samples in proteomic data. **(B)** Proportions of single and multiple phosphorylation sites per DEP in phosphoproteome. **(C)** Overlap of the DEPs identified in different samples of phosphoproteomic data. **(D)** Proportions of different phosphorylated amino acid residues in each sample. **(E)** Predicted subcellular localization of DEPs in proteome and phosphoproteome.

The proteomes and phosphoproteomes of Mock and *AhABI4*s-silenced plants with or without NaCl treatment were also performed (Supplementary Figure 2). TMT-labeling tryptic peptides were fractionated and analyzed by high pH reverse-phase LC-MS/MS. In all eight samples, 9,230 proteins were identified of which 7,748 were quantified (Supplementary Tables 6, 7). Among them, translation levels of 836 proteins (Figure 3A, areas a, b, d, e, f, and l; Supplementary Table 8) were affected by silencing of *AhABI4*s under salt-stress.

For the phosphoproteomics analysis, affinity enrichment was performed with IMAC microspheres before LC-MS/MS. A total of 8,273 phosphorylated sites were detected by spectrum search analysis, 74.79% of them, that is 6,187 sites were filtered out with a localization probability higher than 0.75, 5,901 out of the 6,187 sites were quantifiable (Supplementary Tables 6, 9). Serine (pSer) accounted for 87.83% of the phosphorylated amino acid residues, while 11.71 and 0.46% of the quantifiable sites were threonine (pThr) and tyrosine (pTyr), respectively (Supplementary Figure 6A). Similar results were reported for other eukaryotes (Ye et al., 2015). Comparing with the proteomic results, these residues were assigned to 2,985 quantifiable proteins (Supplementary Table 9). The number of phosphorylation sites and regulation types (up/downregulated) of these sites varied considerably among individual proteins. Up to 87.79% of the phosphorylated proteins had no more than three phosphorylated amino acid residues (Figure 3B). The highest number of phosphorylation sites were identified in a predicted latex abundant protein (arahy.AUQB01) expressed in the roots. Nearly half of the differentially phosphorylated sites (DPSs) were identified only in Mock or *AhABI4*s-silenced plants (Figure 3C, Supplementary Table 10). Though the total number of sites significantly differed among samples, their pSer, pThr, and pTyr distribution ratios were nearly the same, whether upregulated or downregulated (Figure 3D). The foregoing data not only characterized the changes at translation and phosphorylation levels caused by silencing of *AhABI4*s under salt-stress, but also validated the VIGS system in peanuts.

To predict the subcellular localization of differentially expressed or modified proteins, the online program WoLF PSORT (Horton et al., 2007) was used. A total of 3,222 DEPs identified from the proteome and phosphoproteome were considered (Figure 3E, Supplementary Table 11). The greatest DEP proportions were assigned to the nucleus (29.33%), the chloroplast (26.66%), and the cytoplasm (19.52%). However, the proteomic analysis disclosed that 24.78% of the identified DEPs were localized to the cytoplasm followed by the nucleus and the chloroplast (Supplementary Figure 6B, Supplementary Table 11), whereas 52.72% of the differentially modified proteins were localized to the nucleus followed by the cytoplasm and the plasma membrane (Supplementary Figure 6C, Supplementary Table 11). According to the reference genome of the cultivar peanut (Bertioli et al., 2019), the DEPs were spread across all the 20 chromosomes (Supplementary Figure 6D). There were 247 and 254 DEPs locate on chr3 and chr13, respectively, while the number of DEPs located to other chromosomes was <200 (Supplementary Table 12).

### Silencing of *AhABI4*s Triggers Essential Changes in Ion Transporter/Channel Expression Under Salt-Stress

**Figure 4:**
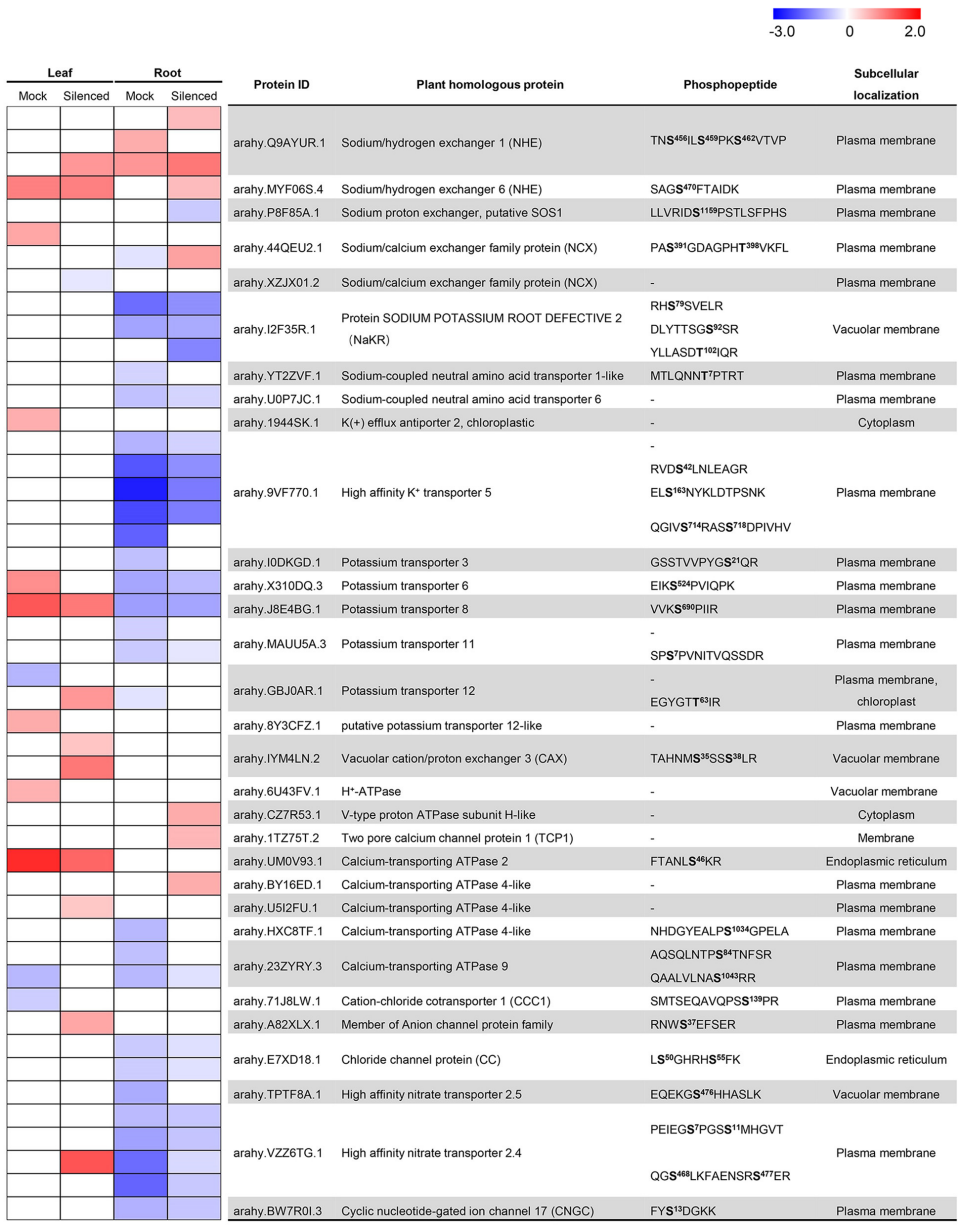
Detection and change ratios of ion transporters and exchangers identified in proteomic and phosphoproteomic data. The color display changing ratio (log_2_ transformed) per protein/site after salt-stress treatment according to the indicated gradient. In the heatmap, the row corresponding to “-” indicated change ratios of the gene at the translational level. Predicted phosphorylation sites marked in bold; superscript indicates the position of modified amino acid in the peptide chain.

Gene ontology analysis was performed using the UniProt-GOA annotation database (www.http://www.ebi.ac.uk/GOA/) to screen for the proteins explaining the phenotypic differences between Mock and *AhABI4*s-silenced plants under salt-stress. The GO enrichment revealed that the DEPs were distributed in 32 GO terms of level 2, including catalytic activity, binding, metabolic process, cellular process, and single-organism process. GO enrichment based on biological process demonstrated that proteins associated with ion transport, ion/cation homeostasis, sodium transport, and regulation of pH were affected by silencing of *AhABI4*s at the protein or phosphorylation level (Supplementary Figures 7A,B). For all samples, 31 proteins participated in ion transport (Figure 4). The previously reported ion transporters SOS1, NHX, sodium/calcium exchanger (NCX), K^+^ efflux antiporter (KEA), H^+^-ATPase, V-type proton ATPase, and CNGC were also identified here. In the present study, *in vivo* phosphorylation of NHX (arahy.IYM4LN.2), NCX (arahy.44QEU2.1, arahy.XZJX01.2), and CNGC (arahy.BW7R0I.3) under salt-stress were first detected (Figure 4). The phosphorylation level of two DPSs in NHX (TAHNM**S**^**35**^SS**S**^**38**^LR) was strongly induced in the leaves of *AhABI4*s-silenced plants under salt-stress. In contrast, no such changes were observed in the Mock plants. Two DPSs (PA**S**^**391**^GDAGPH**T**^**398**^VKFL) were detected in NCX (arahy.44QEU2.1), while upregulated Ser^**391**^ phosphorylation was found only in leaves of Mock plants. Thr^**398**^ was downregulated in roots of Mock plants but upregulated in *AhABI4*s-silenced plants.

Mutation of *NaKR1* (*sodium-potassium root defective 1*) causes accumulation of Na^+^ and K^+^ in leaves (Tian et al., 2010). However, phosphorylation of NaKR proteins under salt-stress has not been reported. In this study, we identified phosphorylated Ser^78^, Ser^82^, and Thr^102^. Of these, only Thr^102^ was strongly reduced in the roots of the *AhABI4s*-silenced plants (Figure 4). Moreover, two potential sodium transporters [sodium-coupled neutral amino acid transporter (SNAT), arahy.YT2ZVF.1, and arahy.U0P7JC.1] were identified. SNAT belongs to the solute carrier protein family in mammals, which mediates the coupled transport of amino acids and Na^+^ across the plasm membrane (Zhang et al., 2009). These results suggested that silencing of *AhABI4*s triggers essential changes of genes that participate in various biological processes, especially the ion transporting, at translation and phosphorylation levels under salt-stress.

### AhABI4s Regulate Ion Homeostasis by Altering Protein Interaction Networks Under Salt-Stress

**Figure 5:**
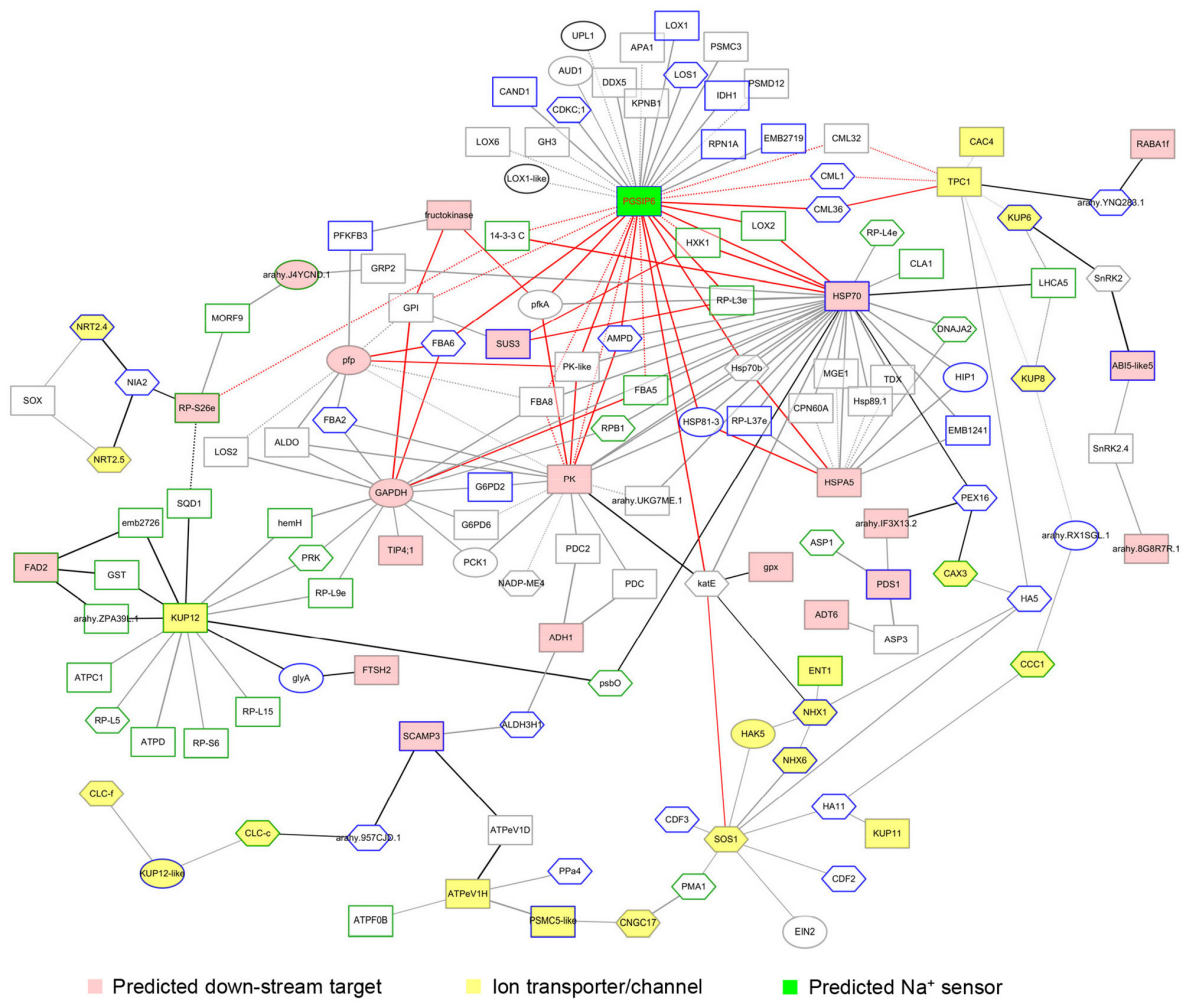
Visualization of the salt-stress response network regulated by AhABI4s. FASTA format of amino acid sequences of identified differentially expressed proteins (DEPs) was submitted to STRING. Interaction databases of *Arabidopsis thaliana, Glycine max*, and *Medicago truncatula* served as references. The minimum required interaction score was >0.7. Certain ion transporters/channels with interaction scores between 0.5 and 0.7 were reserved. Pink and yellow nodes represent predicted downstream targets of AhABI4s and the ion transporters/channels affected by silencing of *AhABI4*s. Board paint of each node indicated the expressed tissue of each protein. Green and gray boards mark proteins expressed in leaves and roots, respectively. Blue boards mark proteins expressed in both leaves and roots. Protein changes at translation, phosphorylation, or both levels represented by rectangle, hexagon, and ellipse, respectively. Widths of edges between nodes represent combined scores.

Sensing high Na^+^ and Cl^−^ concentration and maintaining ion homeostasis in plant cells are the main aspects of plant response to salt-stress (Yang and Guo, 2018a). In this study, we noted that the expression and modification of 31 ion transporters/channels were affected by the silencing of *AhABI4*s under salt-stress. However, the regulatory pathways were unclear. Protein-protein interaction networks were integrated with tissue expression patterns and translation or post-translation levels. It represented a salt-stress response network regulated by *AhABI4*s, which comprised 144 proteins and 214 interactions (Figure 5, Supplementary Table 13). The three proven downstream target genes were localized at important crosslink positions in this network. Downstream targets regulated the function of ion transporter/channel (Figure 5; yellow nodes) *via* interaction with other DEPs (Figure 5; black edges). Expression pattern (expression/phosphorylation level or tissues) of proteins linked by dotted lines in Figure 5 displayed different changing trends or intensity in Mock and *AhABI4*s-silenced plants under salt-stress (Figure 5, Supplementary Figure 8A).

**Figure 6:**
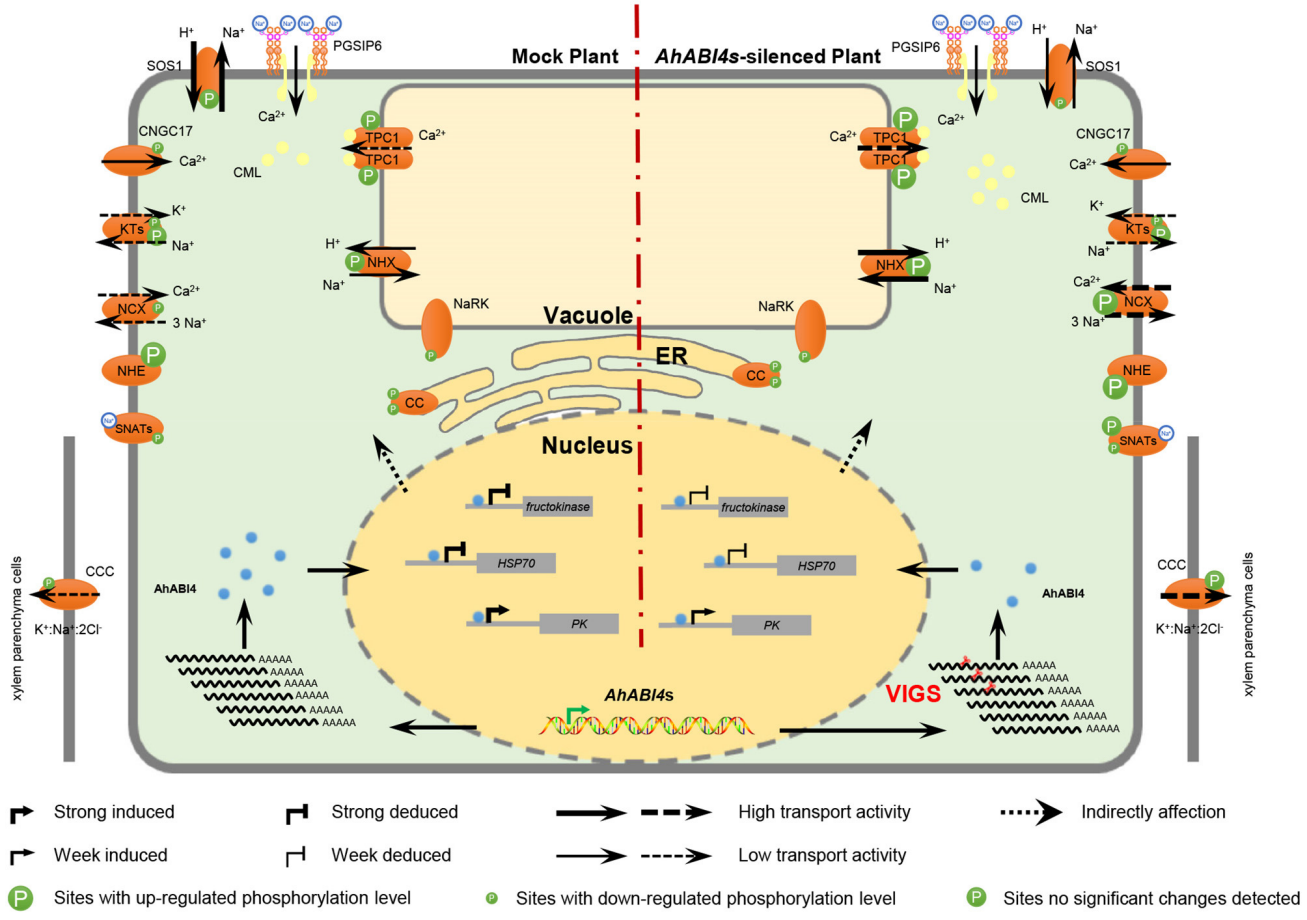
Hypothetical model of ion homeostasis regulated by AhABI4s under salt-stress. Virus-induced gene silencing downregulated *AhABI4*s and influenced the expression of downstream targets (nucleus). Changes in protein levels of the downstream targets altered protein-protein interactions, which further lead to phosphorylation level and phosphorylation sites alteration in ion transporter/channel. Predicted peanut Na^+^ sensor PGSIP6 detected extracellular Na^+^ and gated Ca^2+^ influx channel on the plasma membrane to increase cytoplasmic Ca^2+^ content. On the other hand, PGSIP6 may also induce the transporting of vacuole Ca^2+^ into the cytoplasm via interaction with CMLs. Silencing of *AhABI4*s facilitated this process by promoting CML accumulation and TPC1 phosphorylation. Elevated Ca^2+^ content triggered a cellular defensive response to salt-stress. Na^+^ in the cytoplasm was transported into vacuoles and out of cells by NHX and NCX, respectively. In *AhABI4*s-silenced plants, NHX and NCX phosphorylation levels were higher than those in Mock plants and corresponded to relatively higher ion transport activity. Excess Na^+^ transported out of cytoplasm was transported into xylem parenchyma cells by CCC protein. Comparatively reduced cytoplasmic Na^+^ content in *AhABI4*s-silenced plants explained their salt tolerance phenotype. Solid and broken arrows represent proven and predicted functions, respectively.

Interestingly, neither salt-stress nor silencing of *AhABI4*s influenced the expression levels of peanut PGSIP6 which is homologous of the *Arabidopsis* Na^+^ sensor *MOCA* (Supplementary Figure 9). However, the expression patterns of 35 probable PGSIP6-interacting proteins were affected by silencing of *AhABI4*s under salt-stress (Figure 6, Supplementary Figure 8B). The protein-protein interaction network disclosed that PGSIP6 interacts with the calmodulin-like proteins (CML) 1/32/36, and further participate in the regulation of TPC1 and CAC4 in peanuts (Figure 5, red edges; Supplementary Figure 8B). Furthermore, PGSIP6 may also interact with downstream targets of ABI4, such as HSP70, and PK (Figure 5). Alteration of the protein-protein interaction patterns by differences in expression and phosphorylation level, and expression location under salt-stress influences ion homeostasis in plant cells. *AhABI4s* might be upstream regulators of the Na^+^ sensor PGSIP6 in peanuts.

## Discussion

Soil salinity is one of the main environmental problems affected global agriculture, which reduces crop productivity and leads to economic losses (Shrivastava and Kumar, 2015). The development of salt-tolerance crops is an important approach to solving this problem. Almost all crops show sensitivity to high salinity, but it differs between crops, among the types of cultivar within a species, and depending on the developmental stages (Di Gioia et al., 2018). In this work, we reported that a decrease of TF *AhABI4*s enhanced the salt tolerance, as well as biomass accumulation, of peanut seedlings under salt-stress (Figure 1, Table 1). Besides, BLAST analysis throughout whole genomes of many crops showed that there is only one gene encode peptide containing ABI4 motif in one set of chromosomes in most crops, including peanut (Supplementary Table 1). Therefore, ABI4 is less likely to be functionally redundant with other TFs. By combining this feature with known results, it can be inferred that ABI4 is likely a negative genetic element in salt-stress tolerance, which can be selected as a potential candidate for CRISPR-Cas mediated genome editing to generate ideal germplasms.

Abscisic acid insensitive 4 regulates gene expression by binding to gene promoters. It has been demonstrated that ABI4 protein can bind to the promoter of itself, ABI5, starch branching enzyme (SBE2.2), type-A *Arabidopsis* response regulators (*ARR*s), mitogen-activated protein kinase kinase kinase 18 (*MAPKKK18*), ACC synthase (*ACS4*/*8*) during sugar signaling, seed germination, ethylene biosynthesis, MAPK signaling cascade induced by ABA (Bossi et al., 2009; Dong et al., 2016; Huang et al., 2017; Zhou et al., 2021). The expression of hundreds of genes with ABI4 binding elements was affected by NaCl treatment, and ABI4 binds to promoters of *HKT1;1, RbohD*, and *VTC2* to regulate Na^+^ and ROS accumulation in a plant (Shkolnik-Inbar et al., 2013; Luo et al., 2020). GO analysis of the DEGs in this study has suggested that *AhABI4*s had modulated genes involved in a large functional spectrum of activities and the ABI4-binding motif-containing genes showed both activated or repressed patterns, in agreement with published microarray analyses of ABI4 mutants (Kerchev et al., 2011; Reeves et al., 2011). However, the mechanism of how to carry out two separate functions is currently unknown. The most accepted hypothesis is that ABI4 might interact with one or more other TFs or transcription co-factors to perform distinct biological functions in different tissues or developmental stages (Chandrasekaran et al., 2020). In this study, we found a preference binding between ABI4 protein and ACAT (G/T) GCA motif in promoters of downregulated genes in leaf (Figure 2B). Therefore, it can be inferred that slight differences in the ABI4-binding motif could also determine the transcriptional function of ABI4.

Proteins such as kinases and ion transporters/channels are the executors of plant response to salt-stress. In this study, we showed that silencing of *AhABI4*s also leads to differences in translation and phosphorylation of peanut proteins under salt-stress. Phosphorylation is the most commonly occurring type of post-translational modification. Over one-third of all eukaryotic proteins can be phosphorylated and participate in various biological processes (Vlastaridis et al., 2017; Mergner et al., 2020). Phosphorylation of certain sites significantly alters the activity of key regulators under salt-stress (Ma et al., 2019; Yang et al., 2019). In the present study, 36 phosphorylation sites were detected in 22 ion transporters/channels, and several of the *in vivo* phosphorylation have not been reported before (Figure 4). In animals and humans, the sodium/calcium exchanger (NCX) protein imports Ca^2+^ in exchange for three sodium ions when the cellular Na^+^ concentration rises above a critical level (Wolf et al., 2001; Singh et al., 2015). The only NCX-like protein known in plants maintains Ca^2+^ homeostasis under salt-stress (Wang et al., 2012). However, phosphorylation of NCX in plants is seldom reported. In this study, we found that silencing of *AhABI4*s affected the expression of the NCX proteins arahy.44QEU2 and arahy.XZJX01 and especially phosphorylation of the former under salt-stress. There is also growing evidence that post-translation modification of certain K^+^ transporter regulators significantly changes K^+^ concentration and explains the relative differences in salt tolerance between glycophytes and halophytes (Himabindu et al., 2016). *Arabidopsis* high-affinity K^+^ transporter 5 protein (AtHAK5) maintains high-affinity K^+^ uptake and plant growth under high Na^+^ concentrations (Nieves-Cordones et al., 2010). Nevertheless, AtHAK5 phosphorylation was only detected *in vitro* (Ragel et al., 2015; Brauer et al., 2016). In the present study, we identified nine root-expressed phosphorylation sites in six potassium transporters *in vivo* (Figure 4). Among these, four were normalized to a putative peanut HAK5 protein (arahy.9VF770). Among them, the phosphorylation level of S^42^, S^163^, and S^718^ were affected by silencing of *AhABI4*s under salt-stress.

Protein-protein interaction plays an important role in many biological processes. The protein-protein interaction networks which can be obtained from omics data will be very useful for mechanism analysis of biological processes and prediction of protein functions. In this study, the transcriptome, proteome, and phosphoproteome data before and after salt-stress treatment of *AhABI4s*-silenced and Mock plants were analyzed, and a protein-protein interaction network regulated by *AhABI4*s under salt-stress was constructed. Glycosyl inositol phosphoryl ceramides (GIPCs) were the first Na^+^ sensor identified in plants, which could further gate Ca^2+^ influx channels (Jiang et al., 2019). However, the mechanism underlying GIPC-activated plant Ca^2+^ influx remains unclear. In this study, the protein or phosphorylation level of three probable CML proteins was affected by salt-stress. According to the protein-protein interaction network, the peanut GIPC (PGSIP6, Supplementary Figure 9) may participate in the regulation of Ca^2+^ channel TPC1 *via* PGSIP6-CML-TPC1 (Figure 6) and further influence Ca^2+^ efflux. This protein-protein interaction network not only describes the functions of *AhABI4*s in peanut salt-stress response. but also helps to clarify the intracellular response process after Na^+^-GIPC binding under salt-stress.

In conclusion, this study overviewed the salt tolerance mechanism in peanuts affected by silencing of *AhABI4s* at both translation and post-translational levels. High-throughput data, specifically protein-protein interaction network predictions, has furnished suggestions for subsequent study, especially the Ca^2+^ efflux mechanism under salt-stress and the identification of new proteins and important phosphorylation sites. Moreover, the hypothetical models proposed herein will also help the ion homeostasis regulation mechanism study under salt-stress.

## Data Availability Statement

The datasets presented in this study can be found in online repositories. The names of the repository/repositories and accession number(s) can be found at: https://www.ncbi.nlm.nih.gov/nuccore/MN088829; https://www.ncbi.nlm.nih.gov/nuccore/MN088830; https://www.ncbi.nlm.nih.gov/bioproject/PRJNA560660; https://www.ebi.ac.uk/pride/archive/projects/PXD015090.

## Author Contributions

FL, ZD, and YW conceived the study, designed the experiments, supervised, and complemented the writing. LL, QW, KZ, XZ, RG, and CW performed the experiments and data analysis. LL and QW made the figures and wrote the original article. CZ provided suggestions. All authors discussed the results and commented on the article.

## Funding

This work was supported by the Peanut Seed Industry Project in Shandong Province, China (No. 2020LZGC001 to FL), an earmarked fund for Agriculture Research System in Shandong Province, China (No. SDAIT-04-03 to FL), an earmarked fund for the China Agriculture Research System (CARS-14 to YW), the Key Program of Research and Development in Shandong province (2019GNC106002 to FL), and Postdoctoral Funding of Shandong Agricultural University to LL.

## Conflict of Interest

The authors declare that the research was conducted in the absence of any commercial or financial relationships that could be construed as a potential conflict of interest.

## Publisher's Note

All claims expressed in this article are solely those of the authors and do not necessarily represent those of their affiliated organizations, or those of the publisher, the editors and the reviewers. Any product that may be evaluated in this article, or claim that may be made by its manufacturer, is not guaranteed or endorsed by the publisher.
