# Supplementary material for: AhABI4s Negatively Regulate Salt-Stress Response in Peanut
Source: Front Plant Sci. 2021 Oct 14;12:741641. doi: 10.3389/fpls.2021.741641 (PMC8551806; doi:10.3389/fpls.2021.741641)
Supplement: Supplementary file 1 [file Table_1.DOCX]

| Primer name | Sequence （5’ to 3’） | Description |
| --- | --- | --- |
| AhABI4-F | ATGGAAATTTCTAACCCTAACG | Forward primer of ORF region of ABI4 |
| AhABI4-R | TCAAATTTCTTCAAAATTCAAAC | Reverse primer of ORF region of ABI4 |
| 3’Out | GCTGTCAACGATACGCTACGTAACG | 3’-RACE Abridged universal primer |
| 3’AB-out | CAAGAGAGTTGCATGGTTATGGAAG | 3’-RACE universal forward primer, outer |
| 3’Adaptor | GCTGTCAACGATACGCTACGTAACGGCATGACAGTG(T)_18_ | 3’-RACE Abridged anchor primer |
| 3’AGSP-F | GTTATGGAAGAAGAAATAGTGAATC | 3’-RACE forward primer for ABI4-A, nested |
| 3’BGSP-F | GTTATGGAAGAAGAAGTATTGAATT | 3’-RACE forward primer for ABI4-B, nested |
| 5’-GSP1 | ATTGTGAGGGGGGTTA | First strand cDNA synthesis for 5’-RACE |
| 5’ABGSP-R2 | GCTGCTGGTGTTAGGT | 5’-RACE reverse primer for 1st round PCR |
| 5’ABGSP-R3 | TTGAGAAAGAGTGGGGATAG | 5’-RACE reverse primer for 2nd round PCR |
| AhUKN1-F | AACGAAGACGACGACGAAGAAC | Forward primer of reference control for q-RT PCR |
| AhUKN1-R | GAACAATAGCAACAAGAAGCAAAGC | Reverse primer of reference control for q-RT PCR |
| PDS-F | GGCCAGAAAAAGTCAAGTTTG | Forward primer for pTRV2:*PDS* |
| PDS-R | TGTGGTCTGCACAAATTTCATCAGG | Reverse primer for pTRV2:*PDS* |
| I-f-F | GAATTCTTTGCTTCCTCGCCCTTCC | Forward primer for cloning of “I-fragment” |
| I-f-R | GGATCCGAGGAAGACAATGTTGAATCCACCA | Reverse primer for cloning of “I-fragment” |
| Probe1-F | ATTATATTATCCACGTAGACATTTT | Forward primer for EMSA of HSP70 (P1) |
| Probe1-R | AAAATGTCTACGTGGATAATATAAT | Reverse primer for EMSA of HSP70 (P1) |
| Probe2-F | AATTACATCACCGGTTTACGTGGAT | Forward primer for EMSA of Fructokinase (P2) |
| Probe2-R | ATCCACGTAAACCGGTGATGTAATT | Reverse primer for EMSA of Fructokinase (P2) |
| Probe3-R | TGTATTATCCACGTAAACCGGTGA | Forward primer for EMSA of Fructokinase (P3) |
| Probe3-R | TCACCGGTTTACGTGGATAATACA | Reverse primer for EMSA of Fructokinase (P3) |
| Probe4-F | TCGCCCTACTCGCACCGTCCAATTT | Forward primer for EMSA of PK (P4) |
| Probe4-R | AAATTGGACGGTGCGAGTAGGGCGA | Reverse primer for EMSA of PK (P4) |
| Probe5-F | TGAGGCGCACACCGAAGGCAACGT | Forward primer for EMSA of PK (P5) |
| Probe5-R | ACGTTGCCTTCGGTGTGCGCCTCA | Reverse primer for EMSA of PK (P5) |
| Probe6-F | TGGATGTTCCACGTTGCCTTCGGT | Forward primer for EMSA of PK (P6) |
| Probe6-R | ACCGAAGGCAACGTGGAACATCCA | Reverse primer for EMSA of PK (P6) |
| Probe (+)-F | aataaaaatccaccgaactattatt | Forward primer for EMSA of positive control |
| Probe (+)-R | aataatagttcggtggatttttatt | Forward primer for EMSA of positive control |

**Supplementary Table 1** **Gene specific primers and probes used in this study.**
