# Supplementary material for: AhABI4s Negatively Regulate Salt-Stress Response in Peanut
Source: Front Plant Sci. 2021 Oct 14;12:741641. doi: 10.3389/fpls.2021.741641 (PMC8551806; doi:10.3389/fpls.2021.741641)
Supplement: Supplementary file 2 [file Table_2.DOCX]

**Supplementary Table 2 Analysis of copy number of ABI4 coding genes in plant**

| **Species** | **Ploidy** | **Number of ABI4 motif** | **ID** |
| --- | --- | --- | --- |
| *Arabidopsis thaliana* | Diploid | 1 |  |
| *Oryza sativa* (*japonica* & indica) | Diploid |  |  |
| *Zea mays* | Diploid |  |  |
| *Gossypium hirsutum* | Diploid |  |  |
| *Glycine max* | Diploid |  |  |
| *Grain sorghum* | Diploid |  |  |
| *Cucumis Sativus* | Diploid |  |  |
| *Populus trichocarpa* | Diploid | 2 |  |
| *Solanum tuberosum* | Tetraploid | 2 |  |
| *Brassica napus* | Tetraploid | 3 |  |
| *Triticum aestivum* | Hexaploid | 3 |  |
| *Arachis duranensis* (diploid ancestor of peanut) | Diploid | 1 |  |
| *Arachis ipaensis* (diploid ancestor of peanut) | Diploid | 1 |  |
| Tifrunner (allotetraploid peanut cultivar) | Tetraploid | 1 | arahy.Tifrunner.gnm1.ann1.XJX19T |
| Shitouqi (allotetraploid peanut cultivar) | Tetraploid | 2 | AH07G07970, AH17G06880 |
| Fenghua 2 (allotetraploid peanut cultivar) | Tetraploid | 2 |  |
