# Supplementary material for: AhABI4s Negatively Regulate Salt-Stress Response in Peanut
Source: Front Plant Sci. 2021 Oct 14;12:741641. doi: 10.3389/fpls.2021.741641 (PMC8551806; doi:10.3389/fpls.2021.741641)
Supplement: Supplementary file 3 [file Table_3.DOCX]

**Supplementary Table 3 Plant ABI4s used in multiple alignment**

| **Protein name** | **Species** | **Gene ID/GenBank Accession NO.** |
| --- | --- | --- |
| AhABI4A | *Arachis hypogaea* (Fenghua2) | MN088829 |
| AhABI4B | *Arachis hypogaea* (Fenghua2) | MN088830 |
| AhABI4 | *Arachis duranensis* | Aradu.88AFG.1 |
| AhABI4 | *Arachis ipaensis* | Araip.K8KF4 |
| AhABI4 | *Arachis hypogaea* (Tiffrunner) | arahy..XJX19T.1 |
| AtABI4 | *Arabidopsis thaliana* | AT2G40220.1 |
| AlABI4 | *Arabidopsis lyrata* | Al_scaffold_0004_2621 |
| BnABI4-1 | *Brassica napus* | CDY07614 |
| BnABI4-2 | *Brassica napus* | CDY52427 |
| BnABI4-3 | *Brassica napus* | CDY54991 |
| BrABI4-1 | *Brassica rapa* | Bra000178.1 |
| BrABI4-2 | *Brassica rapa* | Bra004978.1 |
| CsABI4 | *Cucumis sativus* | Csa_6G011730 |
| GmABI4 | *Glycine max* | GLYMA_02G264700 |
| GrABI4 | *Gossypium raimondii* | KJB74386 |
| MtABI4 | *Medicago truncatula* | MTR_5g082950 |
| NaABI4 | *Nicotiana attenuate* | A4A49_08618 |
| OsABI4 | *Oryza sativa L. ssp. indica* | BGIOSGA018232 |
| OsABI4 | *Oryza sativa L. ssp. japonica* | Os05g0351200 |
| PtABI4-1 | *Populus trichocarpa* | PNT23242 |
| PtABI4-2 | *Populus trichocarpa* | PNT17333 |
| StABI4-1 | *Solanum tuberosum* | DMT400092312 |
| StABI4-1 | *Solanum tuberosum* | DMT400085168 |
| SbABI4 | *Sorghum bicolor* | KXG21759 |
| TcABI4 | *Theobroma cacao* | TCM_042471 |
| TaABI4-1 | *Triticum aestivum* | TraesCS1A02G223400.1 |
| TaABI4-2 | *Triticum aestivum* | TraesCS1B02G236700.1 |
| TaABI4-3 | *Triticum aestivum* | TraesCS1D02G225000.1 |
| VvAVI4 | *Vitis Vinifera* | VIT_13s0067g01400 |
| ZmABI4 | *Zea mays* | Zm00001d038001. |
