# Supplementary material for: AhABI4s Negatively Regulate Salt-Stress Response in Peanut
Source: Front Plant Sci. 2021 Oct 14;12:741641. doi: 10.3389/fpls.2021.741641 (PMC8551806; doi:10.3389/fpls.2021.741641)
Supplement: Supplementary file 4 [file Table_4.DOCX]

**Supplementary Table 4 Sequence information of peanut ABI4 coding genes**

| **Gene** | **Full-length cDNA**  **(bp)** | **Length of 5’ UTR**  **(bp)** | **Length of 3’ UTR**  **(bp)** | **Length of CDS (bp)** | **Length of peptide**  **(number of amino acid)** |
| --- | --- | --- | --- | --- | --- |
| *AhABI4A* | 1753 | 349 | 384 | 1092 | 363 |
| *AhABI4B* | 1807 | 312 | 384 | 1074 | 357 |
