# Supplementary material for: AhABI4s Negatively Regulate Salt-Stress Response in Peanut
Source: Front Plant Sci. 2021 Oct 14;12:741641. doi: 10.3389/fpls.2021.741641 (PMC8551806; doi:10.3389/fpls.2021.741641)
Supplement: Supplementary file 6 [file Table_6.DOCX]

**Supplementary Table 6 Overview of differently expressed proteins and phosphorylated sites**

| **Plant Sample** | **Regulation type** |  | **Proteome** |  | **Phosphorproteome** | |
| --- | --- | --- | --- | --- | --- | --- |
|  |  |  | **Number of DEPs** |  | **Number of phosphorylation sites** | **Number of Normalized proteins** |
| Leaf of Mock plant | up |  | 381 |  | 248 | 213 |
|  | down |  | 319 |  | 493 | 375 |
| Leaf of *AhABI4*s-silenced plant | up |  | 107 |  | 279 | 240 |
|  | down |  | 117 |  | 150 | 124 |
| Root of Mock plant | up |  | 506 |  | 487 | 366 |
|  | down |  | 596 |  | 1131 | 788 |
| Root of *AhABI4*s-silenced plant | up |  | 416 |  | 655 | 477 |
|  | down |  | 505 |  | 852 | 611 |
