# Supplementary material for: AhABI4s Negatively Regulate Salt-Stress Response in Peanut
Source: Front Plant Sci. 2021 Oct 14;12:741641. doi: 10.3389/fpls.2021.741641 (PMC8551806; doi:10.3389/fpls.2021.741641)
Supplement: Supplementary file 14 [file Data_Sheet_1.pdf]

Supplementary Figure 1

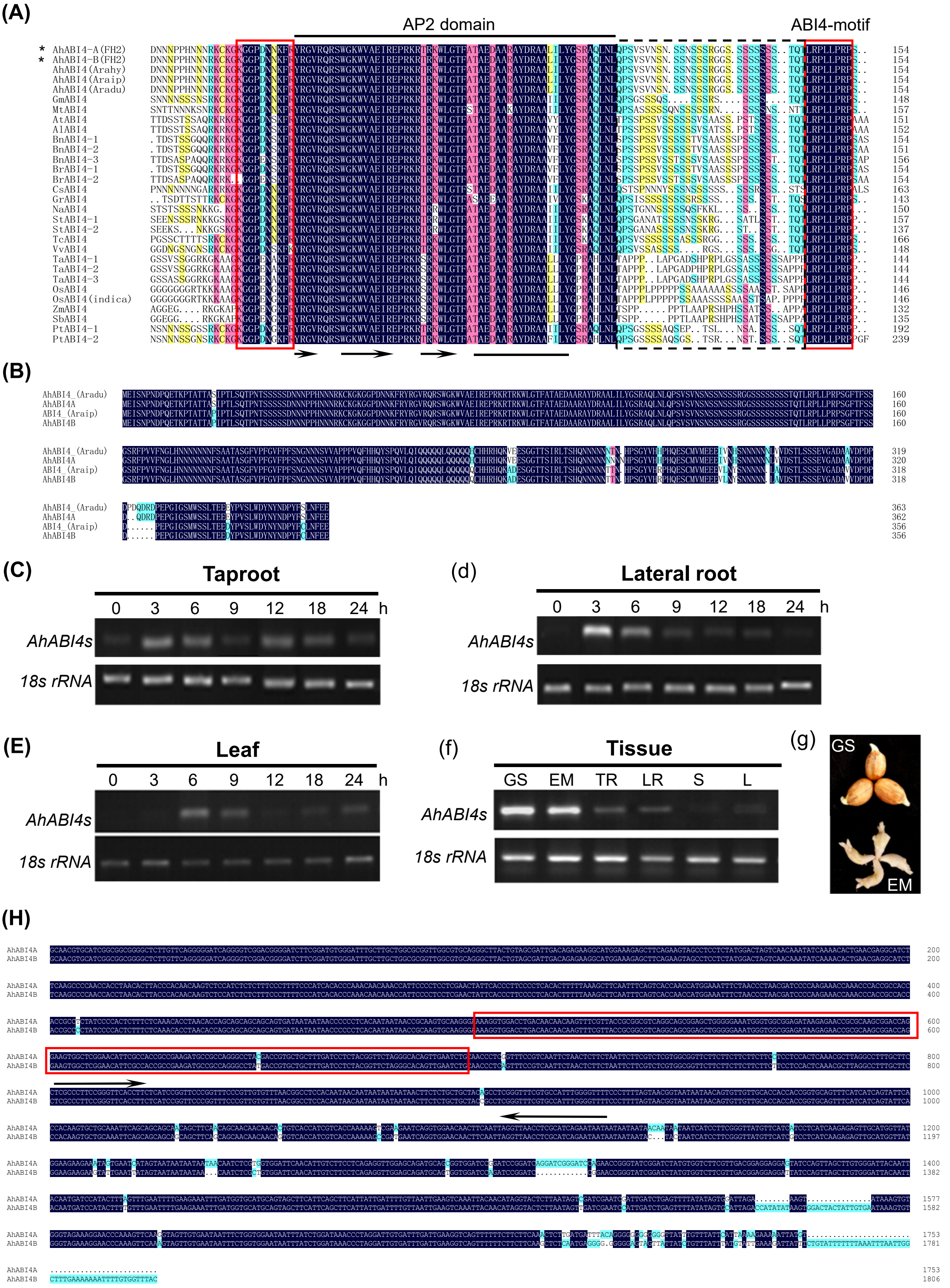

Supplementary Figure 1 Sequence and expression pattern analyses of predicted peanut *ABI4*s. **(A)** Multiple alignment of amino acid sequences of plant ABI4s. **(B)** Alignment of amino acid sequence between the two new cloned ABI4s and ABI4s from *A. duranensis* and *A. ipaensis*. Letters on a dark blue background correspond to identical amino acid residues. **(C-F)** Expression pattern analysis of AhABI4 in different tissues with or without salt stress treatment. TR, taproot; LR, lateral root; S, stem; L, leaf. Three independent replications were performed. At least ten seedlings were harvested at each time point of every repeat. **(G)** Germinating seed (GE) and embryo (EM) used for expression pattern analysis. **(H)** Nucleotide sequences comparison of *AhABI4A* and *AhABI4B*. The fragment encoding AP2-domain and AP2-associated motif is outlined by red box. The forward and reverse primers used for cloning of "I-fragment" are marked by black arrows.
