# Supplementary material for: AhABI4s Negatively Regulate Salt-Stress Response in Peanut
Source: Front Plant Sci. 2021 Oct 14;12:741641. doi: 10.3389/fpls.2021.741641 (PMC8551806; doi:10.3389/fpls.2021.741641)
Supplement: Supplementary file 15 [file Data_Sheet_2.pdf]

Supplementary Figure 2

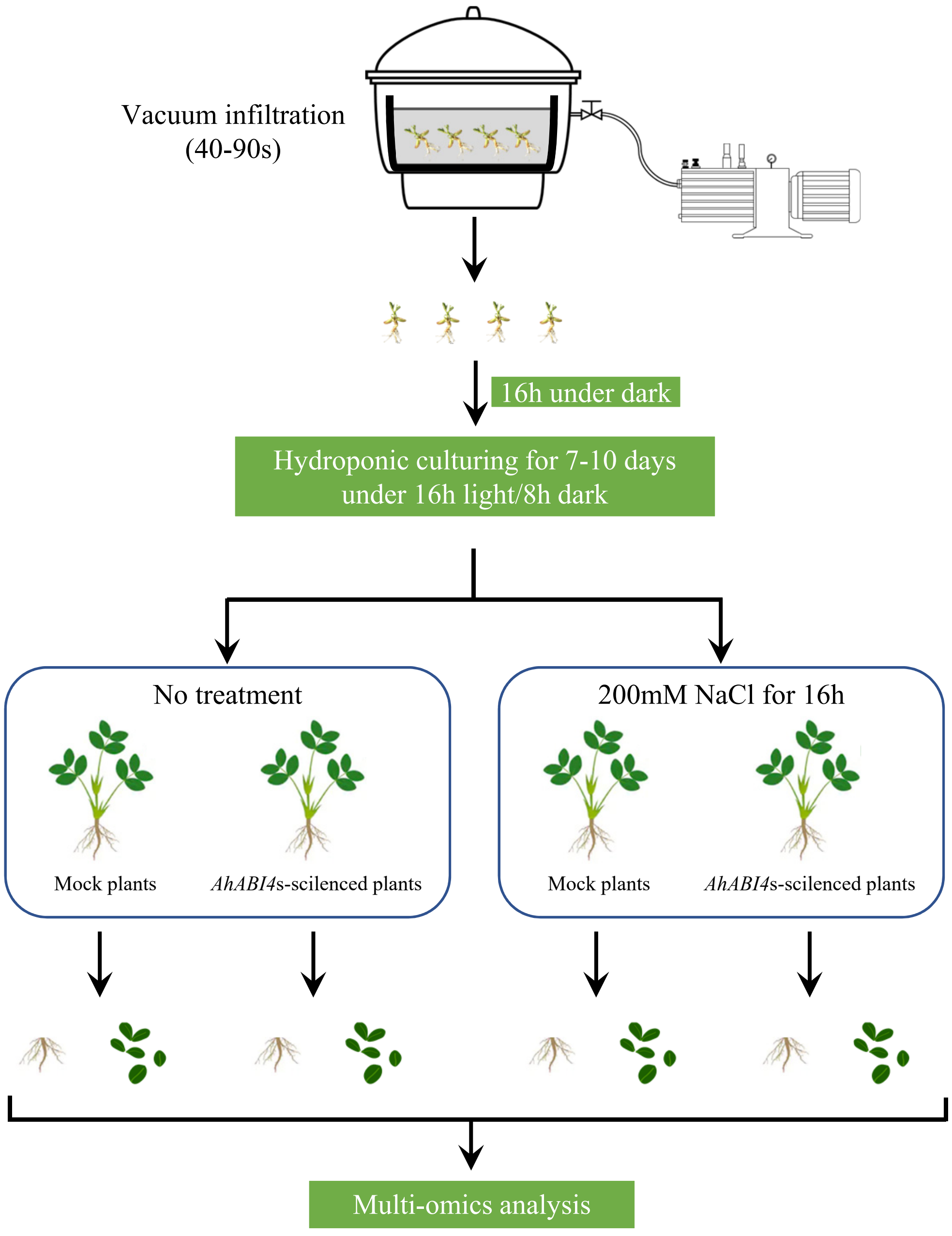

Supplementary Figure 2 Vacuum infiltration VIGS and sample harvest for multi-omics analysis.
