# Supplementary material for: AhABI4s Negatively Regulate Salt-Stress Response in Peanut
Source: Front Plant Sci. 2021 Oct 14;12:741641. doi: 10.3389/fpls.2021.741641 (PMC8551806; doi:10.3389/fpls.2021.741641)
Supplement: Supplementary file 16 [file Data_Sheet_3.pdf]

Supplementary Figure 3

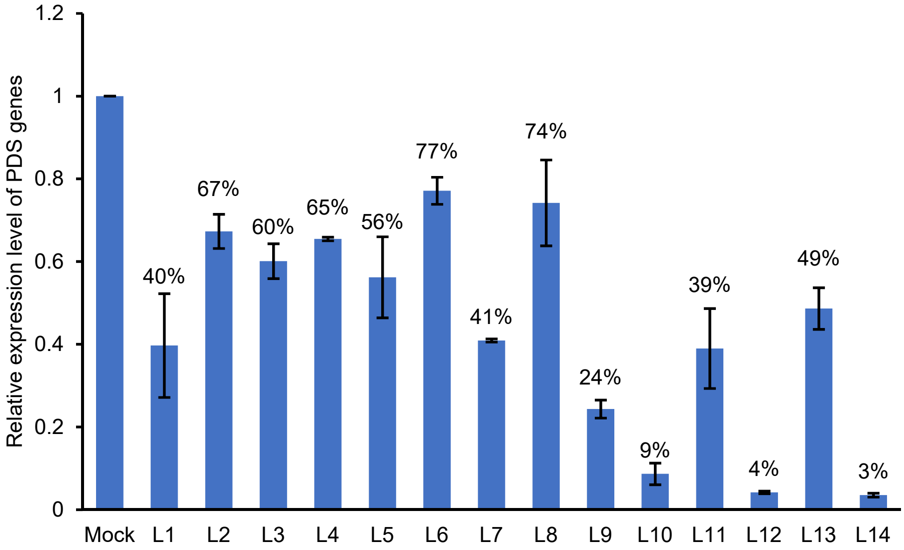

Supplementary Figure 3 Relative mRNA levels of PDS genes in *AhPDS*-silenced lines (L1 to L14) compared with that of Mock plants.
