# Supplementary material for: AhABI4s Negatively Regulate Salt-Stress Response in Peanut
Source: Front Plant Sci. 2021 Oct 14;12:741641. doi: 10.3389/fpls.2021.741641 (PMC8551806; doi:10.3389/fpls.2021.741641)
Supplement: Supplementary file 17 [file Data_Sheet_4.pdf]

Supplementary Figure 4

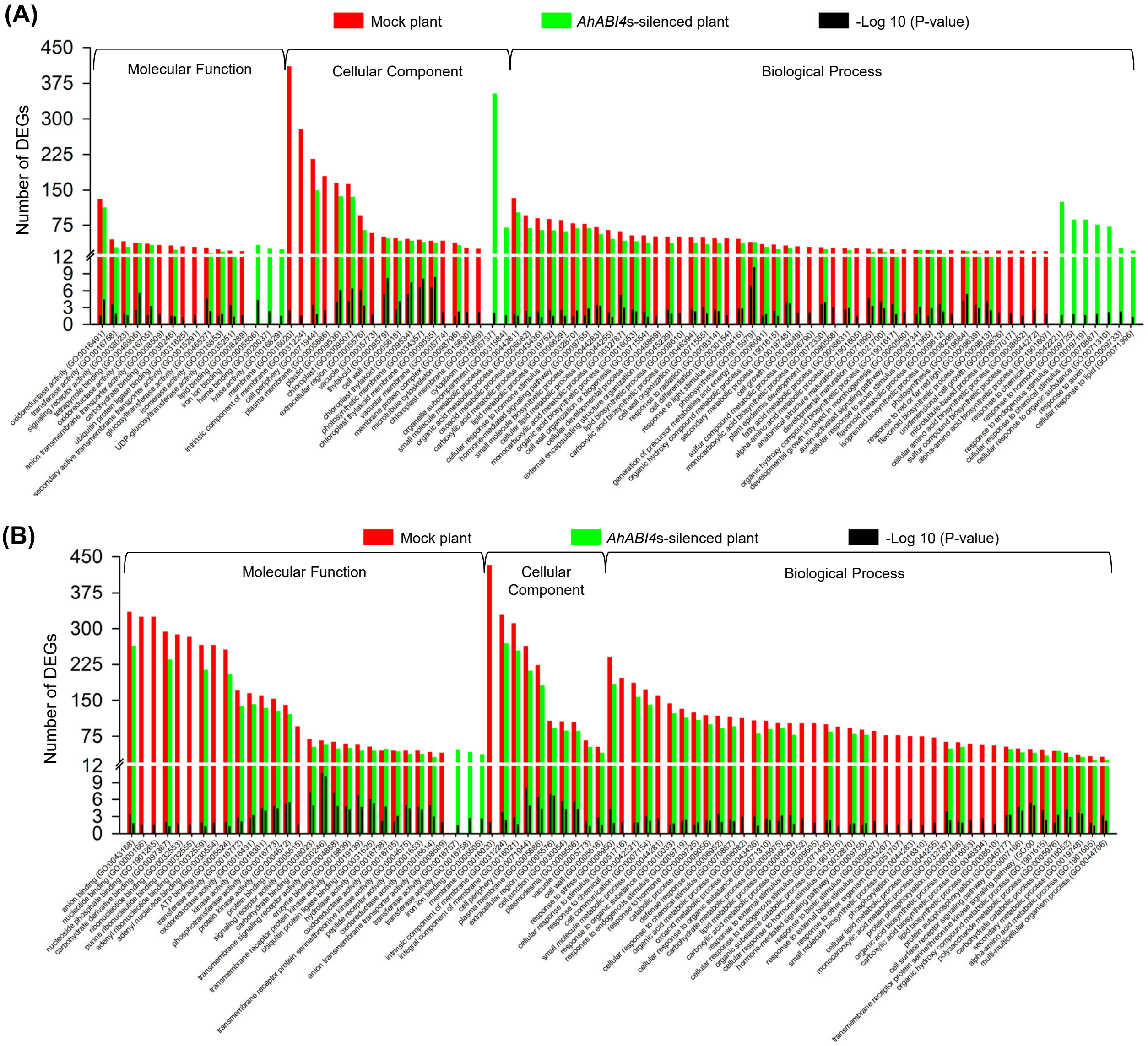

Supplementary Figure 4 GO enrichment of DEGs with S-box in their promoter region. Terms with more than 30 genes in leaf **(A)** and root **(B)** are shown.
