# Supplementary material for: AhABI4s Negatively Regulate Salt-Stress Response in Peanut
Source: Front Plant Sci. 2021 Oct 14;12:741641. doi: 10.3389/fpls.2021.741641 (PMC8551806; doi:10.3389/fpls.2021.741641)
Supplement: Supplementary file 18 [file Data_Sheet_5.pdf]

Supplementary Figure 5

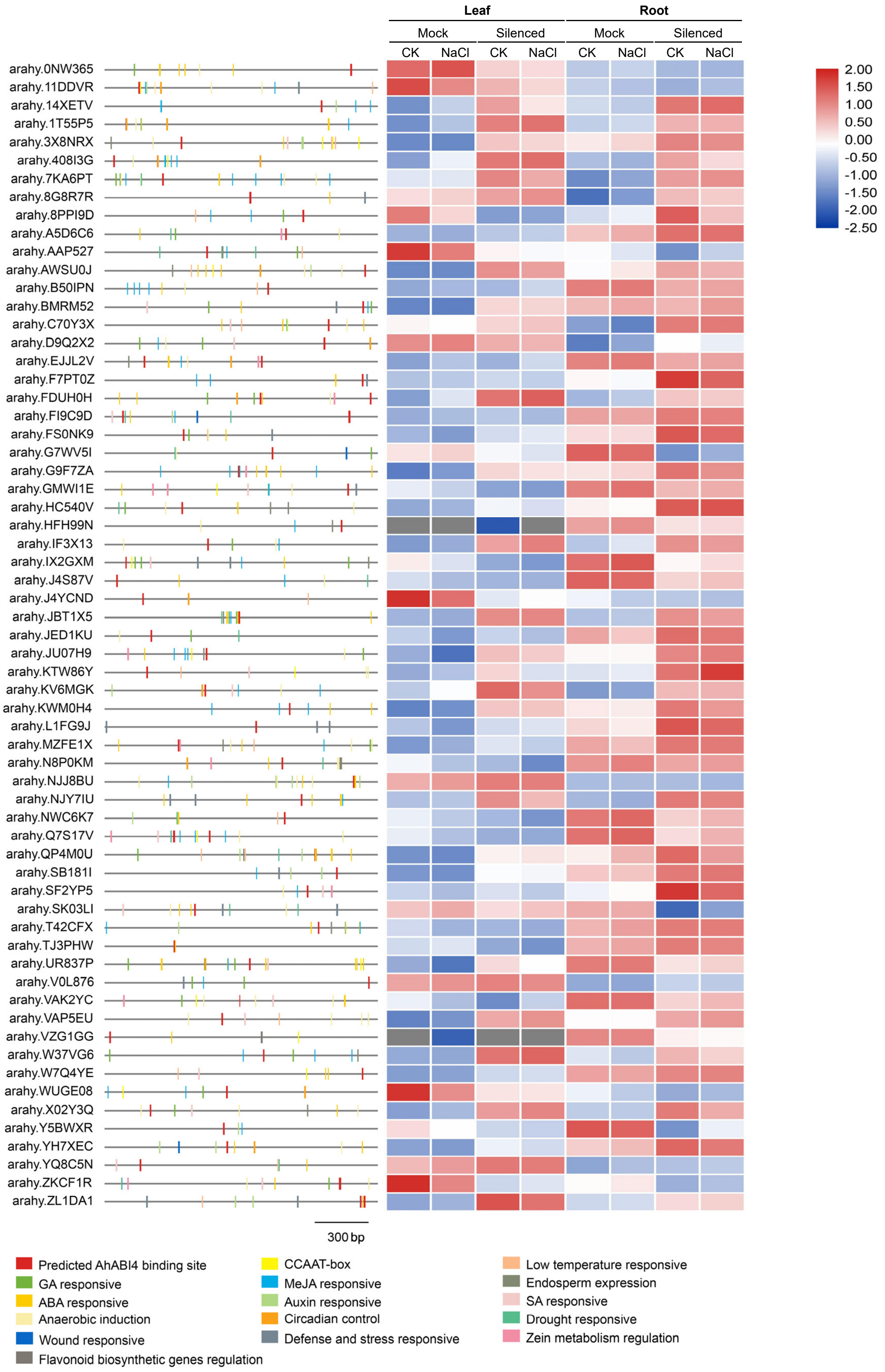

Supplementary Figure 5 The 63 potential down stream targets of AhABI4s under salt stress. The *cis*-elements in promoter (Left) and expression heat map (Right) of potential AhABI4 targets share similar expression in transcription and translation level are showing. The log10 value of FPKM were normalized by row for better visualization.
