# Supplementary material for: AhABI4s Negatively Regulate Salt-Stress Response in Peanut
Source: Front Plant Sci. 2021 Oct 14;12:741641. doi: 10.3389/fpls.2021.741641 (PMC8551806; doi:10.3389/fpls.2021.741641)
Supplement: Supplementary file 19 [file Data_Sheet_6.pdf]

Supplementary Figure 6

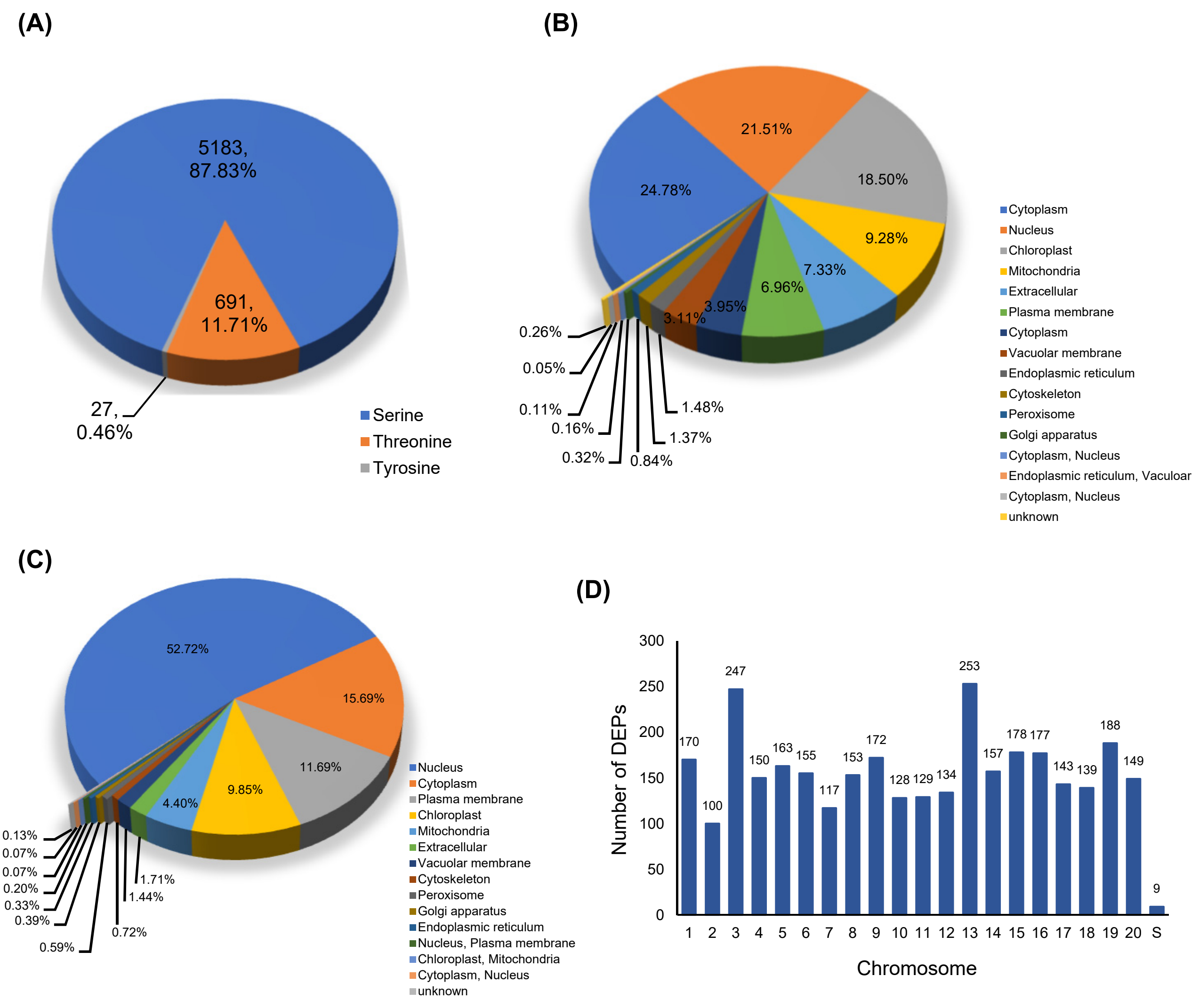

Supplementary Figure 6 Characteristics of proteome and phosphoproteome. **(A)** Distribution of serine, threonine and tyrosine with different modification levels. **(B)** Predicated subcellular of DEPs identified in proteome. **(C)** Predicated subcellular of DEPs identified in phosphoproteome. Proteins with unknown subcellular localization refer to those which were identified in root but predicted to localize in chloroplast. **(D)** Distribution of different expression/phosphorylation proteins among chromosomes. S, stand for scaffold.
