# Supplementary material for: AhABI4s Negatively Regulate Salt-Stress Response in Peanut
Source: Front Plant Sci. 2021 Oct 14;12:741641. doi: 10.3389/fpls.2021.741641 (PMC8551806; doi:10.3389/fpls.2021.741641)
Supplement: Supplementary file 20 [file Data_Sheet_7.pdf]

# Supplementary Figure 7

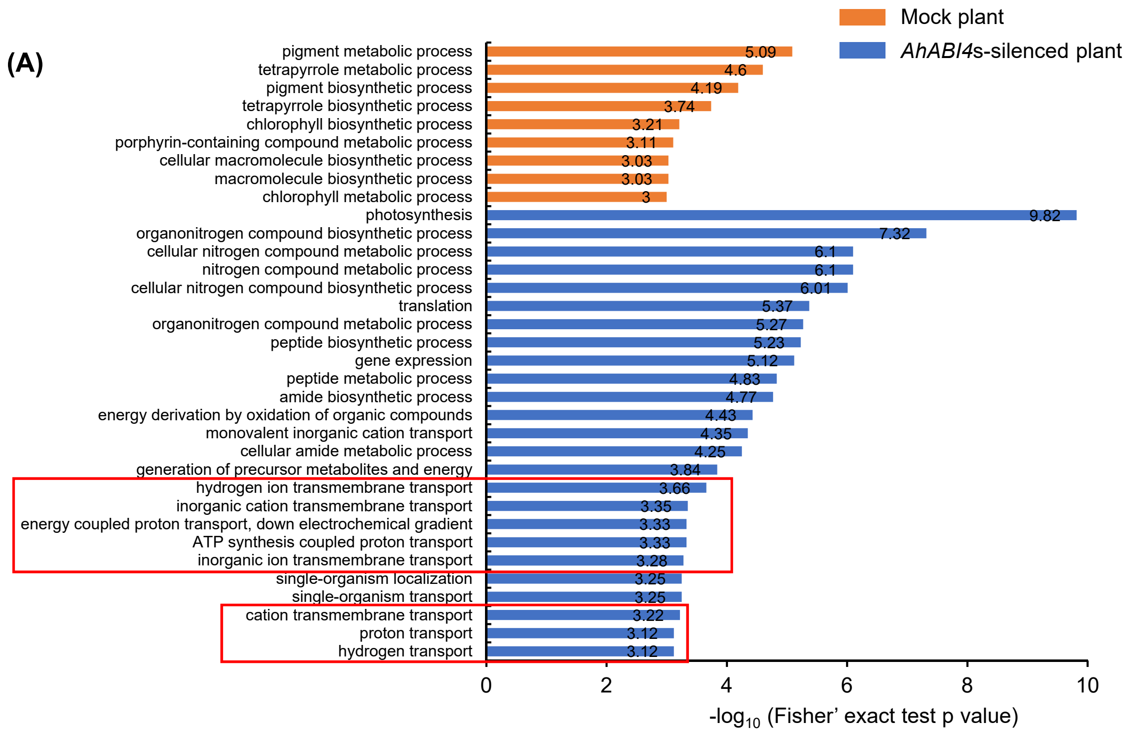


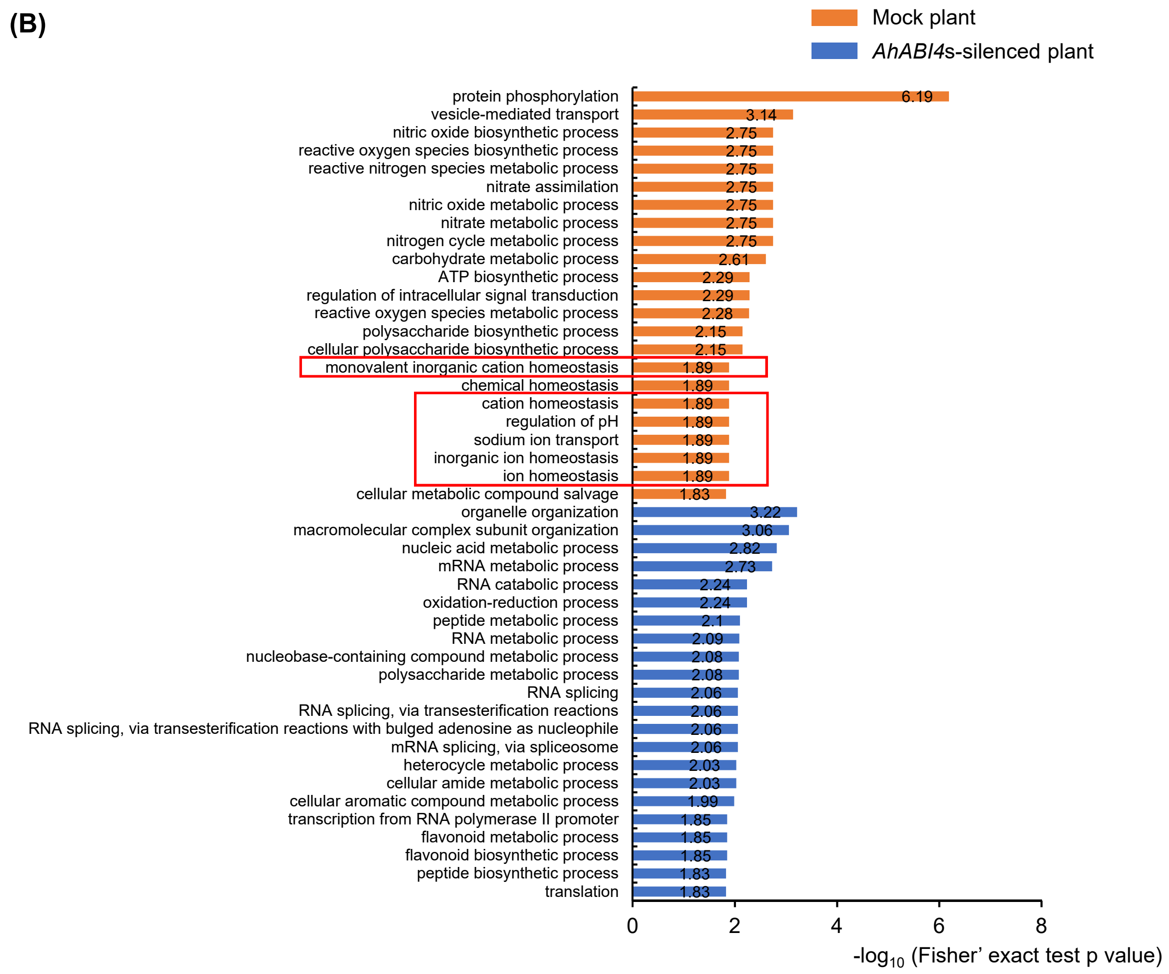


**Supplementary Figure 7** GO enrichment of DEPs in proteome and phosphoproteome (P<0.05). **(A)** GO enrichment of biological progress of differential expression proteins in proteome (partial). **(B)** GO enrichment of biological progress of differential expression proteins in phosphoproteome (partial).
