# Supplementary material for: AhABI4s Negatively Regulate Salt-Stress Response in Peanut
Source: Front Plant Sci. 2021 Oct 14;12:741641. doi: 10.3389/fpls.2021.741641 (PMC8551806; doi:10.3389/fpls.2021.741641)
Supplement: Supplementary file 21 [file Data_Sheet_8.pdf]

Supplementary Figure 8

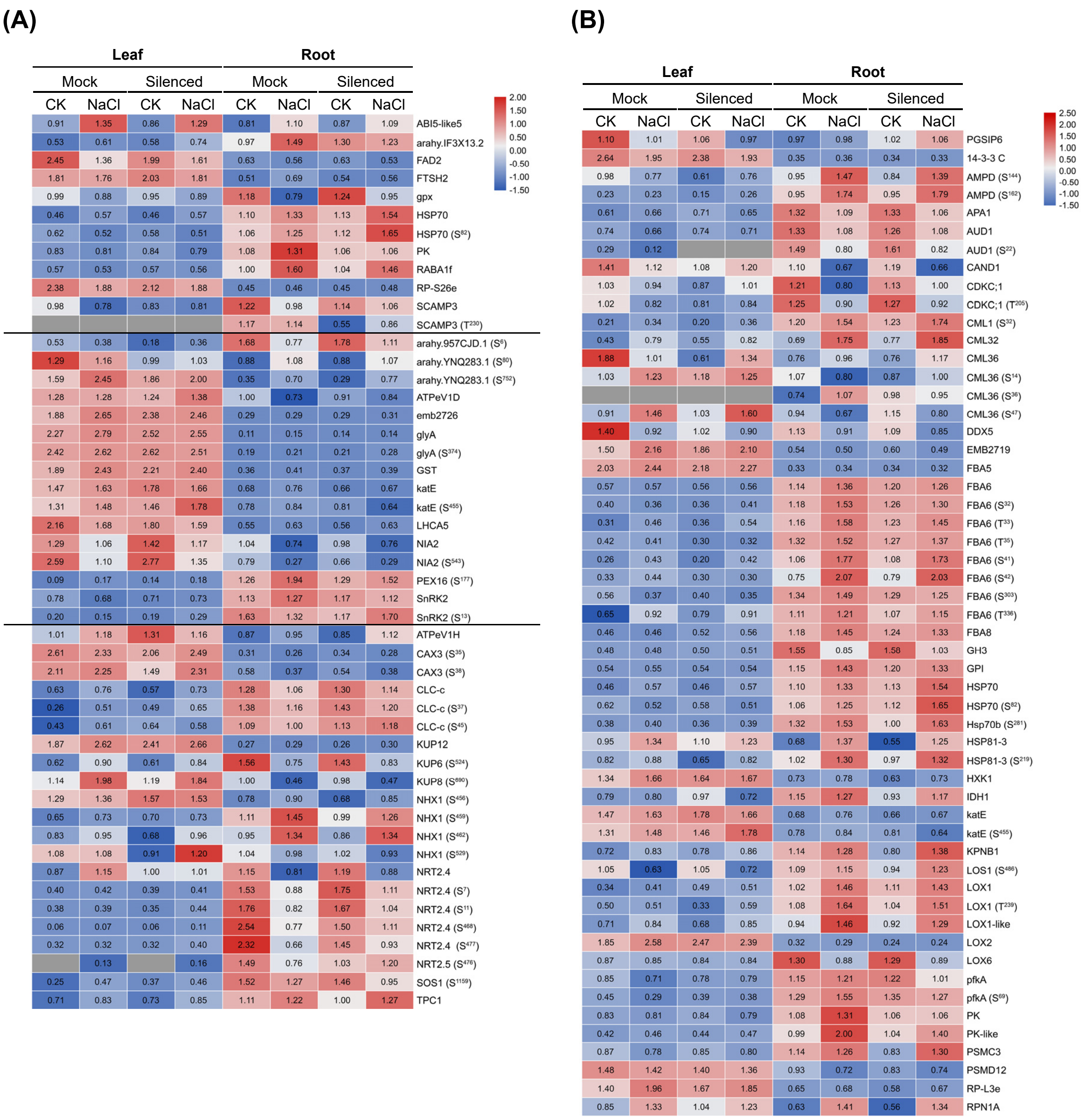

Supplementary Figure 8 Visualization of expression levels of ion transporters/channels, predicted Na^{+} sensor, and their interaction proteins. **(A)** Expression level of down-stream targets (upper), interacted proteins (middle) and the ion transporters/channels in proteome and phosphoproteome. **(B)** Expression level of predicted Na^{+} sensor PGSIP6 and interacted proteins in proteome and phosphoproteome. Average estimated expression levels (log2 transformed) were normalized per row by normal distribution and colored according to the indicated color gradient. The original data were shown in each tile. The legend text showed the power only.
