# Supplementary material for: AhABI4s Negatively Regulate Salt-Stress Response in Peanut
Source: Front Plant Sci. 2021 Oct 14;12:741641. doi: 10.3389/fpls.2021.741641 (PMC8551806; doi:10.3389/fpls.2021.741641)
Supplement: Supplementary file 22 [file Data_Sheet_9.pdf]

Supplementary Figure 9

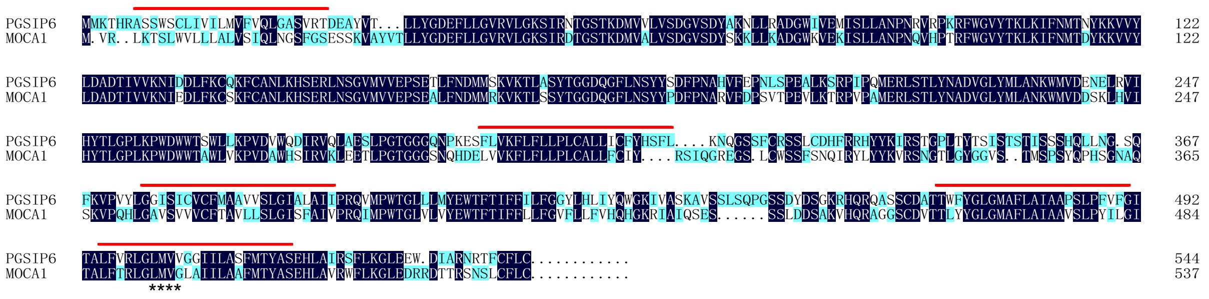

Supplementary Figure 9 Alignment of *Arabidopsis* Na^+ sensor MOCA1 and its homologous gene in peanut. Transmembrane domains were marked by red lines, and the Na^+ binding region was indicated using *.
